# Test–Retest Data for the Assessment of Breast MRI Radiomic Feature Repeatability

**DOI:** 10.1002/jmri.28027

**Published:** 2021-12-22

**Authors:** R.W.Y. Granzier, A. Ibrahim, S. Primakov, S.A. Keek, I. Halilaj, A. Zwanenburg, S.M.E. Engelen, M.B.I. Lobbes, P. Lambin, H.C. Woodruff, M.L. Smidt

**Affiliations:** ^1^ Department of Surgery Maastricht University Medical Centre+ Maastricht The Netherlands; ^2^ GROW – School for Oncology and Developmental Biology, Maastricht University Maastricht The Netherlands; ^3^ Department of Radiology and Nuclear Medicine Maastricht University Medical Centre+ Maastricht The Netherlands; ^4^ The D‐Lab, Department of Precision Medicine Maastricht University Medical Centre+ Maastricht The Netherlands; ^5^ Division of Nuclear Medicine and Oncological Imaging, Department of Medical Physics Hospital Center Universitaire De Liege Liege Belgium; ^6^ Department of Nuclear Medicine and Comprehensive diagnostic center Aachen (CDCA) University Hospital RWTH Aachen University Aachen Germany; ^7^ OncoRay – National Center for Radiation Research in Oncology, Faculty of Medicine and University Hospital Carl Gustav Carus, Technische Universität Dresden, Helmholtz‐Zentrum Dresden Rossendorf Dresden Germany; ^8^ National Center for Tumor Diseases (NCT), Partner Site Dresden, Germany: German Cancer Research Center (DKFZ) Heidelberg Germany; ^9^ Faculty of Medicine and University Hospital Carl Gustav Carus Technische Universität Dresden Dresden Germany; ^10^ Helmholtz Association/Helmholtz‐Zentrum Dresden – Rossendorf (HZDR) Dresden Germany; ^11^ Department of Medical Imaging Zuyderland Medical Center Sittard‐Geleen the Netherlands; ^12^ Health Innovation Ventures Maastricht The Netherlands

**Keywords:** breast, MRI, radiomics, feature repeatability

## Abstract

**Background:**

Radiomic features extracted from breast MRI have potential for diagnostic, prognostic, and predictive purposes. However, before they can be used as biomarkers in clinical decision support systems, features need to be repeatable and reproducible.

**Objective:**

Identify repeatable radiomics features within breast tissue on prospectively collected MRI exams through multiple test–retest measurements.

**Study Type:**

Prospective.

**Population:**

11 healthy female volunteers.

**Field Strength/Sequence:**

1.5 T; MRI exams, comprising T2‐weighted turbo spin‐echo (T2W) sequence, native T1‐weighted turbo gradient‐echo (T1W) sequence, diffusion‐weighted imaging (DWI) sequence using b‐values 0/150/800, and corresponding derived ADC maps.

**Assessment:**

18 MRI exams (three test–retest settings, repeated on 2 days) per healthy volunteer were examined on an identical scanner using a fixed clinical breast protocol. For each scan, 91 features were extracted from the 3D manually segmented right breast using Pyradiomics, before and after image preprocessing. Image preprocessing consisted of 1) bias field correction (BFC); 2) *z*‐score normalization with and without BFC; 3) grayscale discretization using 32 and 64 bins with and without BFC; and 4) *z*‐score normalization + grayscale discretization using 32 and 64 bins with and without BFC.

**Statistical Tests:**

Features' repeatability was assessed using concordance correlation coefficient(CCC) for each pair, i.e. each MRI was compared to each of the remaining 17 MRI with a cut‐off value of CCC > 0.90.

**Results:**

Images without preprocessing produced the highest number of repeatable features for both T1W sequence and ADC maps with 15 of 91 (16.5%) and 8 of 91 (8.8%) repeatable features, respectively. Preprocessed images produced between 4 of 91 (4.4%) and 14 of 91 (15.4%), and 6 of 91 (6.6%) and 7 of 91 (7.7%) repeatable features, respectively for T1W and ADC maps. *Z*‐score normalization produced highest number of repeatable features, 26 of 91 (28.6%) in T2W sequences, in these images, no preprocessing produced 11 of 91 (12.1%) repeatable features.

**Data Conclusion:**

Radiomic features extracted from T1W, T2W sequences and ADC maps from breast MRI exams showed a varying number of repeatable features, depending on the sequence. Effects of different preprocessing procedures on repeatability of features were different for each sequence.

**Level of Evidence:**

2

**Technical Efficacy Stage:**

1

The use of radiomics to answer diagnostic, predictive, and prognostic questions has increased in recent years, especially in the field of oncology.^1^ Radiomics refers to the extraction of large amounts of high‐throughput quantitative data from medical images using mathematical algorithms that have the potential to noninvasively reveal more information about the region of interest than can be captured by visual inspection alone.^2^ The extracted quantitative data, termed radiomics features, capture information regarding the shape, intensity, and texture of the chosen region of interest (ROI), which is usually the lesion or the affected organ. Radiomics features are intended to serve as biomarkers for the development of clinical decision support systems to enhance personalized medicine.^3^

In breast cancer research, multiple radiomics studies have shown promising results for diagnostic, prognostic, and predictive purposes.^4^, ^5^, ^6^ Despite these seemingly promising results, translation to clinical practice is limited.^7^ A major translational bottleneck can be attributed to the often unknown effect that multiple steps in the radiomics workflow have on feature values, including image acquisition, reconstruction, and preprocessing.^8^, ^9^, ^10^, ^11^ For a radiomics feature to serve as a biomarker, and to be used reliably in clinical decision support systems, it must fulfill the criteria *repeatability* and *reproducibility*.^12^ Repeatability can be defined as “the variability of the biomarker when repeated measurements are acquired on the same experimental unit under identical or nearly identical conditions” and reproducibility as to “variability in the biomarker measurements associated with using the imaging instrument in real‐world clinical settings, which are subject to a variety of external factors that cannot all be tightly controlled.”^12^

Previous research has already identified several steps in the radiomics workflow that influence the reproducibility and repeatability of radiomics features. For example, image acquisition and reconstruction appear to cause variation in radiomic feature values in research performed on CT imaging.^13^, ^14^ Unlike the Hounsfield Units in CT, MRI does not have absolute signal intensities, potentially causing large differences between images, emphasizing the importance of inspecting and possibly adjusting image intensities before performing feature extraction.^15^ A test–retest MRI study of glioblastoma showed that both normalization and intensity quantization strategies affect radiomic feature repeatability and that the optimal strategy must be composed per feature group.^16^ Further test–retest studies assessing feature repeatability have been performed in cervical,^17^ and prostate cancer^18^, ^19^ and have shown consistent results, although all studies state that translation of results to other tumor sites has not been confirmed. In contrast, Peerlings et al^20^ showed that 9.2% (122/1322) of the features, extracted from apparent diffusion coefficient (ADC) maps in ovarian, liver, and colorectal cancer patients, were repeatable among the different tumor sites.

The assessment of radiomics feature repeatability by test–retest studies in breast MRI exams is currently lacking. A potential reason for this lack of data is the variance present in a standard clinical breast MRI protocol, which means that scanning parameters may differ between patients scanned with the same clinical protocol. Therefore, this study investigated the repeatability of radiomics feature values extracted from breast MRI exams using a fixed clinical breast protocol comprising of T2‐weighted (T2W) images, T1‐weighted (T1W) images, and diffusion‐weighted images (DWI) and their derived ADC maps.

## Material and Methods

### Study Population

The study was approved by the local medical ethical committee and written informed consent was given by all participants before participation. Eleven healthy female volunteers were recruited via college‐wide advertisement. Participants were only included if they did not suffer from claustrophobia and met the requirements for admission to the MRI. Participants' height, weight, and the phase of the menstrual cycle were noted. The menstrual cycle of the included healthy volunteers was not taken into account during the MRI exams.

### Imaging Acquisition

All MRI exams were performed using a 16‐channel breast coil on one single 1.5 T scanner (Ingenia, Philips Healthcare, Best, The Netherlands) in the same research institution by the same technician. During imaging, the women lay in the prone position with both breasts in the openings of the breast coil and both arms above their head. The performed MRI protocol consisted of a T2‐weighted turbo spin echo (T2W), native T1‐weighted turbo gradient echo (T1W), and a single shot diffusion‐weighted imaging (DWI) sequence using b‐values of 0, 150, and 800. A single corresponding ADC‐map was derived from all three DWI sequences. All volunteers underwent MRI exams using the identical breast protocol while maintaining as many parameters fixed as possible. The acquisition parameters for the different MRI sequences are shown in the supplementary material (Table S1). The shimbox, needed for the T1W and DWI sequences, was placed on the sternum by default. In case the technician judged the scan as clinically insufficient, the shimbox was placed on the breasts.

### Study Design

**FIGURE 1:**
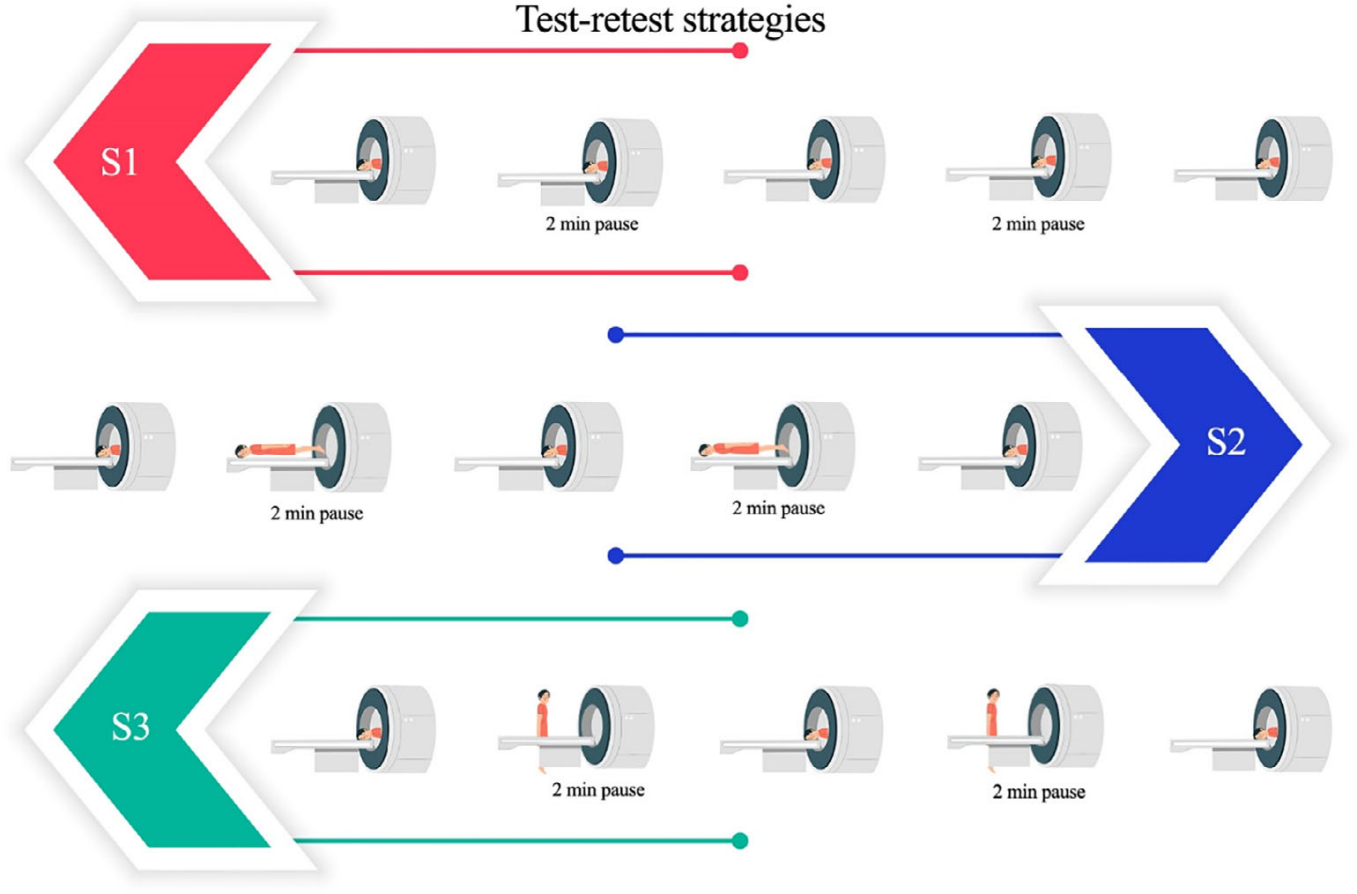
Visual representation of the three test–retest strategies.

A test–retest study was designed to assess the repeatability of breast‐MRI extracted radiomic features. Three separate test–retest strategies were performed twice at 6–10 day intervals. From here on, we will use ‘date 1’ to refer to the first scanning date of each healthy volunteer and ‘date 2’ to refer to the second scanning date. In each strategy, the complete breast MRI protocol was repeated three times with a 2‐minute pause between each protocol. In the first strategy (S1) the participant remained in the MRI scanner the entire time (including the pauses) without movement, for the acquisition of the three breast MRI protocols. The second strategy (S2) differed from S1 only by moving the table out of the scanner (with the participant still in the same position without movement) during the 2‐minute breaks. For the third strategy (S3) the participant got off the table during the 2 minutes breaks (Fig. 1). In total, 18 different MRI exams were acquired for each healthy volunteer with a total scanning time of approximately 198 minutes.

### ROI Segmentation

**FIGURE 2:**
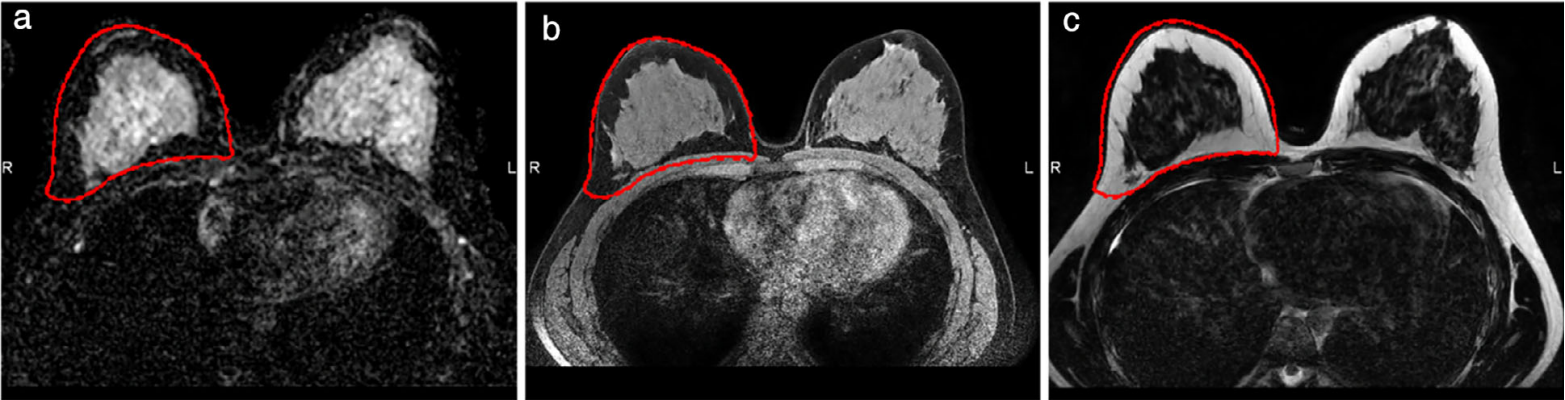
An axial slice of a 3D MRI exam of a healthy volunteer including right breast segmentation (red margin). **(a)** ADC map, **(b)** T2‐weighted image, **(c)** T1‐weighted image

All images were visually checked for quality(including artifacts) by a dedicated breast radiologist with 14 years of experience (ML) before starting the analysis. The region of interest (ROI) was segmented by a medical researcher (RG) with 4 years of experience in breast MR imaging and validated by the same dedicated breast radiologist. It was chosen to 3D, manually segment the right breast. The segmentations were bounded by the sternum (medial side), the pectoral muscle (dorsal side), and the axilla (lateral side) in three dimensions using MIM software (version 7.1.3, Cleveland, OH, USA). Segmentations were performed on all patients on the T2W sequences of all MRI exams as anatomical structures are best visible on this sequence. Subsequently, the T2W sequence was registered with the T1W sequence, and ADC map, using rigid alignments within MIM software, followed by segmentations transfer (Fig. 2).

### Image Preprocessing and Feature Extraction

All MRI exams including ROI segmentations were converted to the nearly raw raster data (NRRD) file format using Python (version 3.7.3) for subsequent analysis. Before feature extraction, multiple preprocessing procedures were applied to the images to study their impact on feature repeatability. First, feature extraction was performed without any image preprocessing as a baseline measurement. Second, N4 bias field correction was applied to the images prior to feature extraction.^21^ Lastly, the bias field corrected images were further preprocessed using the built‐in image *z*‐score normalization by Pyradiomics software (version 2.2.0), with and without binning the voxel grayscale values using a fixed bin width of 32 and 64 (Pyradiomics suggested a bin width between 16 and 128).^16^, ^22^ Image preprocessing steps were performed in Python (version 3.7) using an in‐house developed pipeline based on the computer vision packages, including OpenCV (version 4.1.0), SimpleITK (version 1.2.0), and NumPy (version 1.16.2). For each ROI, 91 original features were extracted using the Pyradiomics software (version 3.0.1), which is mostly compliant with the Image Biomarker Standardization Initiative.^23^ The extracted radiomics feature included first‐order statistics features, gray‐level co‐occurrence matrix features (GLCM), gray‐level run length matrix features (GLRLM), gray‐level size zone matrix features (GLSZM), neighboring gray tone difference matrix features (NGTDM), and gray‐level dependence matrix features (GLDM). All texture features were extracted using default Pyradiomics settings. A detailed Pyradiomics feature description can be found online.^24^

### Statistical Analysis

To assess the repeatability of the extracted radiomic features for the various ROI's in the multiple test–retest strategies, the concordance correlation coefficient (CCC) was calculated using the epiR package (Version 0.9‐99) (REF) in R language (version 3.6.3) performed in R studio (version 1.2.1335, Vienna, Austria).^25^ Radiomics features extracted from a given MRI exam are compared to radiomic features extracted from the remaining MRI exams in a pairwise manner. The CCC was used to evaluate the agreement in radiomic feature values, taking into account both the rank and the value of the measurements.^26^ This metric has the advantage of robust results in small sample sizes.^26^ The CCC provides values between −1 and 1, with 0 representing no concordance, 1 representing perfect concordance, and −1 perfect inverse concordance. Features with a CCC of >0.90 were defined as repeatable features, according to suggestions in literature.^27^ Feature concordance was assessed for each preprocessing procedure using the results of all test–retest strategies of both scanning dates as well as for the results collected on the separate scanning dates. To create an overview of repeatable features across all pairs for the different preprocessing procedures, the intersection of the repeatable features across pairs was noted.

## Results

### Patients Demographics

The median age of the 11 healthy female volunteers was 28 years (interquartile range 25–30 years). Table 1 summarizes the healthy volunteers' characteristics. Shimbox displacement occurred in 22.6% of the scanned sequences.

**TABLE 1:** Patient Characteristics

	Healthy Volunteers (*n* = 11)
Age (years) (median; IQR)	28 (25–30)
Height (cm) (median; IQR)	167 (167–172)
Weight (kg) (median; IQR)	60 (58–63)
Week of the menstrual cycle^a^	Date 1/date 2
Week 1	1/5
Week 2	1/1
Week 3	3/1
Week 4	4/2
Days between scan (mean; range)	7 (6–9)

IQR: interquartile range.

^a^ No measurement of the menstrual cycle possible for two healthy volunteers.

### Repeatable Radiomic Features

**FIGURE 3:**
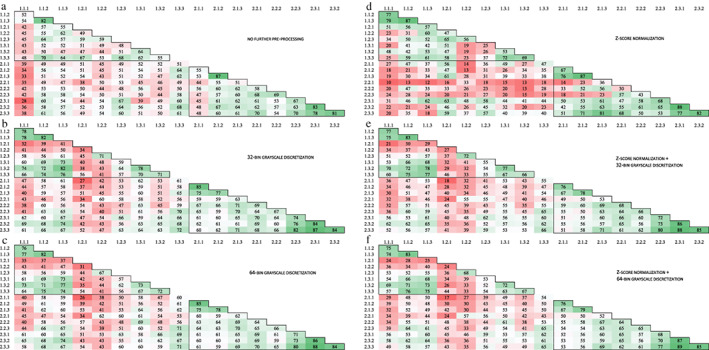
Number of pairwise concordant radiomic features using a concordance correlation coefficient > 0.90 for T1‐weighted images with **(a)** no further preprocessing, **(b)** 32‐bin grayscale discretization, **(c)** 64‐bin grayscale discretization, **(d)** *z*‐score normalization, **(e)** *z*‐score normalization +32‐bin grayscale discretization, and **(f)** *z*‐score normalization +64‐bin grayscale discretization. The black frame in the top left corner shows the MRI exams taken during the first scan date and the black frame in the bottom right corner shows the MRI exams taken during the second scan date. The numbers on the axis refer to the different MRI exams scanned, wherein the first number corresponds to the scan date and the second number to the test–retest strategy. In each test–retest strategy, three scans were examined which is represented by the last number. A total of 91 radiomic features was examined.

**FIGURE 4:**
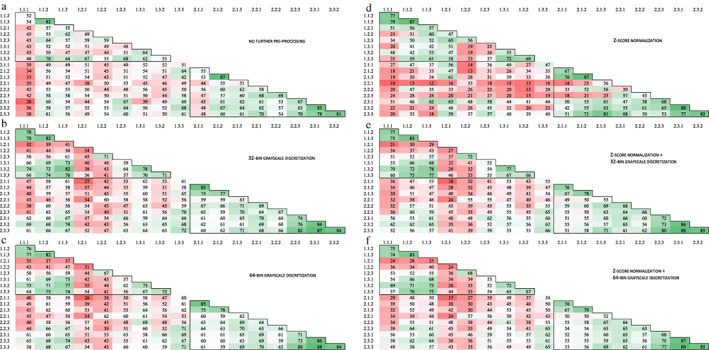
Number of pairwise concordant radiomic features using a concordance correlation coefficient >0.90 for T2‐weighted images with **(a)** no further preprocessing, **(b)** 32‐bin grayscale discretization, **(c)** 64‐bin grayscale discretization, **(d)** *z*‐score normalization, **(e)** *z*‐score normalization +32‐bin grayscale discretization, and **(f)** *z*‐score normalization +64‐bin grayscale discretization. The black frame in the top left corner shows the MRI exams taken during the first scan date and the black frame in the bottom right corner shows the MRI exams taken during the second scan date. The numbers on the axis refer to the different MRI exams scanned, wherein the first number corresponds to the scan date and the second number to the test–retest strategy. In each test–retest strategy, three scans were examined which is represented by the last number. A total of 91 radiomic features was examined.

**FIGURE 5:**
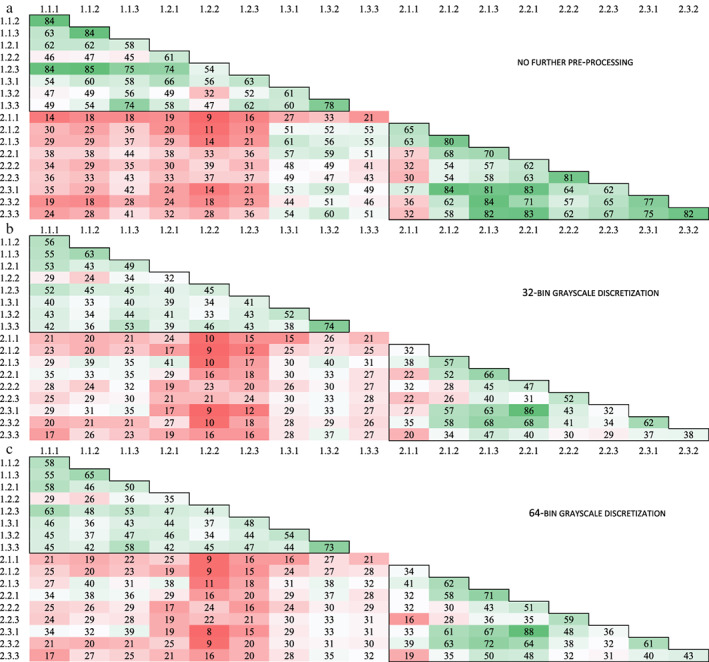
Number of pairwise concordant radiomic features using a concordance correlation coefficient >0.90 for ADC maps with **(a)** no further preprocessing, **(b)** 32‐bin grayscale discretization, and **(c)** 64‐bin grayscale discretization. The black frame in the top left corner shows the MRI exams taken during the first scan date and the black frame in the bottom right corner shows the MRI exams taken during the second scan date. The numbers on the axis refer to the different MRI exams scanned, wherein the first number corresponds to the scan date and the second number to the test–retest strategy. In each test–retest strategy, three scans were examined which is represented by the last number. A total of 91 radiomic features was examined.

Due to a scanning error of all T1‐weighted images and the ADC maps of one healthy volunteer during scanning date 1, all data of this participant was excluded from the analysis. In both the T1W and T2W sequences as in the ADC maps, in pairwise comparison, the number of concordant features varied per scanning date, per test–retest strategy and, per image preprocessing procedure (Figs. 3, 4, 5). Furthermore, for all preprocessing procedures, the lowest number of concordant features was observed between the MRI exams scanned on date 1 and the MRI exams scanned on date 2, seen in the reddest field outside the black demarcations in Figs. 3, 4, 5.

### T1W Sequence

Across all pairs, regardless of scanning date and test–retest strategy, the highest number of concordant features was seen in the images without preprocessing, resulting in 15 of 91 (16.5%) concordant features. These 15 features consisted of 7 first‐order, 1 GLCM, 2 GLRLM, 2 GLSZM, and 2 GLDM and, 1 NGTDM feature(s) (Table 2). Applying grayscale discretization resulted in 13 of 91 (14.3%) and 14 of 91 (15.4%) concordant features for 32‐bins and 64‐bins, respectively. Compared to the images without preprocessing, the texture features showed less concordant features. The *z*‐score normalized images resulted in the lowest number of 4 of 91 (4.4%) concordant features. Applying gray‐scale discretization after *z*‐score normalization improved the number of concordant textural features to 7of 91 (7.7%) and 8 of 91 (8.8%) for 32‐bins and 64‐bins, respectively. The loss in the number of concordant features for *z*‐score normalized images (with and without grayscale discretization), when compared to the images without preprocessing, was mainly due to a loss in the number of concordant first‐order features, which were 6 of 91 (6.6%).

**TABLE 2:** Concordant Features across All Pairs for the T1‐Weighted MRI Exams, with A: No Preprocessing, B: 32‐Bin Grayscale Discretization, C: 64‐Bin Grayscale Discretization, D: *z*‐score Normalization, E: *z*‐score Normalization +32‐bin Grayscale Discretization, and F: *z*‐score Normalization +64‐bin Grayscale Discretization

	A	B	C	D	E	F
Number of Concordant Features	15 (16.5%)	13 (14.3%)	14 (15.4%)	4 (4.4%)	7 (7.7%)	8 (8.8%)
firstorder_90Percentile	×	×	×			
firstorder_InterquartileRange	×	×	×			
firstorder_MeanAbsoluteDeviation	×	×	×			
firstorder_Mean	×	×	×			
firstorder_RobustMeanAbsoluteDeviation	×	×	×			
firstorder_RootMeanSquared	×	×	×			
firstorder_Skewness	×	×	×	×	×	×
glcm_JointAverage	×					
glrlm_GrayLevelNonUniformity	×	×	×		×	×
glrlm_RunLengthNonUniformity	×	×	×		×	×
glszm_GrayLevelNonUniformity	×		×	×		×
glszm_SizeZoneNonUniformity				×		
glszm_SmallAreaHighGrayLevelEmphasis	×					
gldm_DependenceNonUniformity	×	×	×		×	×
gldm_GrayLevelNonUniformity	×	×	×	×	×	×
ngtdm_Busyness		×	×		×	×
ngtdm_Coarseness	×	×	×		×	×

For the majority of preprocessing strategies, the images collected during date 2 showed a higher number of concordant features (varying between 10/91 and 48/91 in images without BFC and between 11/91 and 35/91 in BFC images) compared to images collected during date 1 (varying between 4/91 and 32/91 in images without BFC and between 9/91 and 14/91 in BFC images) (Table 3, Fig. 3), with these differences being greatest after applying grayscale discretization. Furthermore, for most image preprocessing procedures, the addition of BFC resulted in less concordant features compared to the images without BFC (Table 3, Table S2 in the Supplemental Material). For the BFC images without further preprocessing and for the BFC images with grayscale discretization, it was mainly the first‐order features that showed a loss of concordance compared to not performing BFC.

**TABLE 3:** Number of Concordant Features Across all Pairs for the Entire Dataset (All) and Across All Pairs from the Separate Scanning Dates (Date 1 and Date 2) for All Sequences With and Without Bias Field Correction, With A: No Further Preprocessing, B: 32‐bin Grayscale Discretization, C: 64‐bin Grayscale Discretization, D: *z*‐score normalization, E: *z*‐score Normalization +32‐bin grayscale discretization, and F: *z*‐Score Normalization +64‐bin Grayscale Discretization

Sequences	Without BFC			With BFC		
	All	Date 1	Date 2	All	Date 1	Date 2
T1W						
A	15	32	40	8	13	11
B	13	19	45	10	11	30
C	14	18	48	8	12	31
D	4	4	10	4	9	12
E	7	10	35	10	13	34
F	8	9	38	8	14	35
T2W						
A	11	31	16	0	1	60
B	7	9	12	2	3	22
C	7	9	11	1	3	23
D	26	35	44	26	39	37
E	4	7	7	6	11	17
F	4	7	6	5	11	18
ADC						
A	8	28	22	8	9	12
B	7	15	13	6	9	12
C	6	11	11	6	11	11

Figures S1–S6 in the Supplemental Material present the pairwise CCC values in scatterplots for all features in the different preprocessing procedures, wherein the different colors represent the use of all pairwise comparisons or only the pairwise comparisons between MRI exams scanned on the same day.

### T2W Sequence

Across all pairs, regardless of scanning date and test–retest strategy, the *z*‐score normalized images showed the highest number of concordant features, 26 of 91 (28.6%), of which, 3 first‐order, 11 GLCM, 3 GLRLM, 0 GLSZM, 8 GLDM, and 1 NGTDM feature(s) (Table 4). Compared to the other preprocessing procedures, the difference in the number of concordant features was mainly in the concordant texture features, which were almost nonconcordant for the other preprocessing procedures.

**TABLE 4:** Concordant Features Across All Pairs for the T2‐weighted MRI Exams, With A: No Preprocessing, B: 32‐bin Grayscale Discretization, C: 64‐bin Grayscale Discretization, D: *z*‐score Normalization, E: *z*‐score Normalization +32‐bin Grayscale Discretization, and F: *z*‐score Normalization +64‐bin Grayscale Discretization

	A	B	C	D	E	F
Number of Concordant Features	11 (12.1%)	7 (7.7%)	7 (7.7%)	26 (28.6%)	4 (4.4%)	4 (4.4%)
firstorder_10Percentile				×	×	×
firstorder_90Percentile	×	×	×			
firstorder_InterquartileRange	×	×	×	×	×	×
firstorder_MeanAbsoluteDeviation	×	×	×			
firstorder_Mean	×	×	×			
firstorder_RobustMeanAbsoluteDeviation	×	×	×	×	×	×
firstorder_RootMeanSquared	×	×	×			
glcm_JointAverage	×					
glcm_Contrast				×		
glcm_DifferenceAverage	×			×		
glcm_DifferenceEntropy				×		
glcm_DifferenceVariance				×		
glcm_JointEntropy				×		
glcm_Idm				×		
glcm_Idmn				×		
glcm_Id				×		
glcm_Idn				×		
glcm_InverseVariance				×		
glcm_SumEntropy				×		
glrlm_GrayLevelNonUniformity				×		
glrlm_RunLengthNonUniformity	×					
glrlm_RunPercentage				×		
glrlm_RunVariance				×		
gldm_DependenceEntropy				×		
gldm_DependenceNonUniformity				×		
gldm_DependenceNonUniformityNormalized				×		
gldm_DependenceVariance				×		
gldm_GrayLevelNonUniformity				×		
gldm_LargeDependenceEmphasis				×		
gldm_LargeDependenceHighGrayLevelEmphasis				×		
gldm_SmallDependenceHighGrayLevelEmphasis				×		
gldm_SmallDependenceLowGrayLevelEmphasis	×	×	×		×	×
ngtdm_Complexity				×		
ngtdm_Contrast	×					

The images without preprocessing resulted in 11 of 91 (12.1%) concordant features across all pairs, of which more than half of these features were first‐order features (Table 4). Applying grayscale discretization resulted in a further decrease of concordant features to 7 of 91 (7.7%) for both 32 and 64 bins. Applying grayscale discretization after *z*‐score normalization resulted in a loss of almost all concordant textural features when compared to *z*‐score normalized images alone. These images resulted in only 4 of 91 (4.4%) concordant features for both 32 and 64 bins. Notably, the only concordant texture feature (gldm_SmallDependenceLowGrayLevelEmphasis) was not concordant after *z*‐score normalization alone.

The addition of BFC resulted in different feature concordance when compared to the same image preprocessing procedures without BFC (Table 4, Table S3 in the Supplemental Material). The BFC images without further preprocessing, with 32‐bin grayscale discretization and, with 64‐bin grayscale discretization resulted in 0 of 91 (0.0%), 2 of 91 (2.2%), and 1 of 91 (1.1%) concordant features, respectively. Despite the overall loss of concordant features, 2 of 91 (2.2%) features were found to be concordant after the addition of BFC. The BFC *z*‐score normalized images showed the same number of concordant features compared to the *z*‐score normalized images without BFC, although some features improved in concordance, where others lost concordance. The application of grayscale discretization after *z*‐score normalization on BFC images showed the same pattern in concordant features when compared to the images without BFC, namely, a loss of almost all concordant textural features (Tables 4 and S3 in the Supplemental Material). These preprocessing procedures resulted in 6 of 91 (6.6%) and 5 of 91 (5.5%) concordant features, for 32‐bins and 64‐bins, respectively. Furthermore, it is noteworthy that when looking at the pairwise concordance features for the different scan dates, BFC decreased the feature concordance for MRI exams scanned on date 1, while there was an increase in feature concordance for MRI exams scanned on date 2 (Fig. 4, Table 3).

Figures S7–S12 in the Supplemental Material present the pairwise CCC values in scatterplots for all features in the different preprocessing procedures, wherein the different colors represented the use of all pairwise comparisons or only the pairwise comparisons between MRI exams scanned on the same day.

### ADC Map

Across all pairs, regardless of scanning date and test–retest strategy, the number of concordant features for the images without preprocessing, with 32‐bin grayscale discretization, and 64‐bin grayscale discretization was 8 of 91 (8.8%), 7 of 91 (7.7%), and 6 (6.6%), respectively (Table 5). In none of the preprocessing procedures, first‐order features appeared to be concordant. The number of concordant features was roughly the same for the BFC images with 8 of 91 (8.8%), 6 of 91 (6.6%), and 6 of 91 (6.6%) concordant features for images without further preprocessing, with 32‐bin grayscale discretization, and 64‐bin grayscale discretization, respectively (Table 5). Although compared to the images without BFC, some features improved in concordance, where others lost concordance (Table 5).

**TABLE 5:** Concordant Features Across All Pairs for the ADC Maps, With A: No Preprocessing, B: 32‐bin Grayscale Discretization, and C: 64‐bin Grayscale Discretization, D: Bias Field Correction, E: Bias Field Correction +32‐bin Grayscale Discretization and, F: Bias Field Correction +64‐bin Grayscale Discretization

	A	B	C	D	E	F
Number of Concordant Features	8 (8.8%)	7 (7.7%)	6 (6.6%)	8 (8.8%)	6 (6.6%)	6 (6.6%)
glcm_ClusterProminence	×					
glcm_Correlation	×	×	×	×	×	×
glcm_Imc1	×	×	×	×		×
glcm_Imc2	×	×	×	×	×	×
glrlm_GrayLevelNonUniformity		×	×		×	×
glrlm_RunLengthNonUniformity	×	×	×	×	×	×
glszm_GrayLevelNonUniformity	×	×	×	×	×	×
glszm_SizeZoneNonUniformity	×			×		
gldm_DependenceNonUniformity	×			×		
ngtdm_Coarseness		×		×	×	

The number of concordant features differed between the images collected on the separated scanning dates, although these differences were minor compared to the T1W and T2W sequences (Fig. 5, Table 3). The number of concordant features was 28 of 91 (30.8%), 15 of 91 (16.5%) and 11 of 91 (12.1%) for date 1 and 22 of 91 (24.1%), 13 of 91 (14.3%) and 11 of 91 (12.1%) for date 2, using the images without BFC. The number of concordant features was 9 of 91 (9.9%), 9 of 91 (9.9%) and 11 of 91 (12.1%) for date 1 and 12 of 91 (13.2%), 12 of 91 (13.2%) and 11 of 91 (12.1%) for date 2, using the BFC images.

Figures S13–S15 in the Supplemental Material present the pairwise CCC values in scatterplots for all features in the different preprocessing procedures, wherein the different colors represented the use of all pairwise comparisons or only the pairwise comparisons MRI exams scanned on the same day.

## Discussion

In this test–retest study, repeatable radiomics features extracted from breast MRI exams from healthy volunteers were identified, using a fixed scanning protocol including T2‐weighted (T2W), unenhanced T1‐weighted (T1W), and diffusion‐weighted images with corresponding derived ADC maps. This study showed the effects of varying image preprocessing procedures on the radiomics feature repeatability. Across all pairs, the images without preprocessing produced the highest number of repeatable features for both the T1W sequence as well as the ADC maps. In the T2W images, applying *z*‐score normalization produced the highest number of repeatable features.

The assessment of radiomics feature repeatability via test–retest studies in breast MRI exams is currently lacking. The three different MRI sequences examined in this study showed differences in feature repeatability. In addition, the effect of image preprocessing on feature repeatability was different for the two MRI sequences and ADC maps. Not applying image preprocessing produced the highest number of repeatable features in the T1W sequence and the ADC maps. Overall, applying grayscale discretization caused a loss of repeatable textural features in the T1W and T2W sequences, although some texture features became repeatable after grayscale discretization. It is notable that in general, the number of repeatable texture features was reduced after applying grayscale discretization, although grayscale discretization is considered necessary for the extraction of texture features by both Pyradiomics and the IBSI guidelines.^22^ Given that MR images do not contain absolute signal values, MRI exams performed on the same scanner using an identical scan protocol could potentially eliminate the need for grayscale discretization. Furthermore, *z*‐score normalized images showed the highest number of repeatable features in the T2W sequence, on the other hand, applying normalization decreased the number of repeatable features in the T1W sequence. Failure to improve the repeatability of features after *z*‐score normalization was also found in the study by Schwier et al,^19^ although, in contrast to our results, this was seen in the T2W sequence. They state that image normalization was used to homogenize images acquired from different scanners with different protocols. In our study, however, it was assumed that images scanned with the same protocol on the same scanner were already well comparable in terms of imaging parameters. In addition, the applied normalization uses the whole image for normalization and since the MRI quality decreases further from the coil (at the edges of the images), this reduction in quality can degrade the quality of the breast region (which is close to the coil) and with that the ROI comparability. The same principle could account for the use of BFC since for all sequences it either did not change the number of repeatable features or caused a loss of repeatable features compared to not using BFC. However, failure to improve the repeatability of functions after BFC may also be due to use of default settings for the N4 BFC; findings of Saint Martin et al^28^ showed that the default settings for the N4 BFC were not optimal for breast MRI exams.

By considering pairwise comparisons between scans taken on the same day, it was found that for all sequences, including all different preprocessing procedures, except for the T2W sequence and ADC maps without preprocessing, date 2 produced a higher number of repeatable features compared to date 1. One explanation for this may be that the healthy volunteers knew better what to expect on the second scan date after going through the first scan date. In addition, in most cases, the number of repeatable features was higher for the scans taken on the same day compared to the number of repeatable features found from the data of both days, as expected. These differences may be explained by changing factors over time (eg, system changes in the MRI scanner or biology of the healthy volunteer) that caused variation in the feature values. For example, the homogeneity of the MRI field, gradient systems, and coil affects the image quality.^29^ Furthermore, changes in the biology of the healthy volunteer, including the menstrual cycle and body temperature, are known to affect the MRI exams.^30^ These factors may impact clinical decision making and hence, radiomic features must be robust to these changes.

To date, MRI test–retest studies for the evaluation of repeatable and reproducible features, have been conducted through phantom research^15^, ^28^, ^31^, ^32^, ^33^ and by the use of MRI exams of healthy volunteers or cancer patients.^17^, ^19^, ^20^, ^32^, ^34^, ^35^, ^36^ None of these studies investigated feature repeatability and/or reproducibility in human breast MRI exams, and only one study investigated a breast phantom.^28^ The study of Saint Martin et al^28^ showed the necessity of image preprocessing dedicated to breast MRI exams before using features in further analysis. Phantom repeatability and reproducibility results seem to be overly optimistic as these overall appear to score higher than the test–retest studies performed within human data. For example, the study by Lee et al^32^ tested feature repeatability in T1W and T2W in both a phantom and MRI brain of healthy volunteers. The average ICC repeatability measures for the T1W and T2W images were higher for the phantom (0.963 and 0.959) compared to healthy volunteers (0.856 and 0.849). Furthermore, a recently published phantom study by Shur et al^31^ showed that 37 of 46 (80%) of the radiomic features were concordant (CCC > 0.9) in a test–retest study. By contrast, the test–retest study by Eck et al^34^ investigating feature repeatability in T2W brain MRI exams of 15 healthy volunteers showed only 76 of 146 (52%) of good to excellent repeatable features (CCC ≥0.7). Considering only the excellent repeatable features (CCC > 0.85) in the above‐mentioned article, the number of concordant features decreased to 40 of 146 (27.4%), which is more comparable to the results found in this study. The same accounts for a test–retest study in brain MRI exams of glioblastoma patients, in which they identified 386 of 1043 (37.0%) repeatable features, although they used CCC > 0.8 as a cut‐off value.^36^ A prostate MRI repeatability study by Schwier et al^19^ concluded that feature repeatability can vary greatly among the radiomic features and that the repeatability of the features is highly sensitive to image preprocessing procedures.

In clinical (prospective) trials, variance in scanners and acquisition and reconstruction parameters between and even within patients is unsurmountable and will therefore affect the reproducibility of the features. Although exploring feature reproducibility was not the aim of this study, this data will be a starting point to investigate the reproducibility of breast MRI extracted radiomic features. Future studies can investigate feature reproducibility by changing the different acquisition parameters one by one while leaving the others fixed. Furthermore, the harmonization method called ComBat, which was originally developed to harmonize gene expression data,^37^ is increasingly being applied in radiomics studies to remove batch effects.^8^, ^14^, ^38^, ^39^, ^40^ However, caution should be exercised when applying this harmonization method, as it can only correct for one variable and, MRI data collected from multiple hospitals often contains a multitude of variables. In addition, future studies should focus on the discriminative power of a repeatable and reproducible feature, as a repeatable and reproducible feature does not necessarily imply that this feature is a predictive or prognostic radiomic feature.

### Limitations

First, the number of healthy volunteers included was quite limited, although the test–retest set‐up allowed for 18 MRI exams per healthy volunteer, resulting in the analysis of a total of 198 MRI exams. Nevertheless, since this is an early study investigating this topic, we believe that these results are valuable and useful for the radiomics community. Second, the included T1W images were examined without adding a contrast agent, so these images cannot be fully compared to the dynamic T1W images normally examined in a clinical breast protocol. Future test–retest studies in breast cancer patients should show whether the repeatable features found in this study are also repeatable in dynamic T1W images. Third, this study investigated only Pyradiomics features extracted from the original image. Future studies could focus more on other feature groups, among others, Gabor, gradient, or Laws. Fourth, the region of interest contained only healthy tissue, further research in breast cancer patients will have to show whether the repeatable features found in healthy breast tissue can also be considered repeatable in breast tumor tissue. Lastly, it is important to keep in mind that there is a great variety of preprocessing procedures, which can influence feature values. In this study, we choose to use the open‐source software Pyradiomics to apply normalization and grayscale discretization to easily reproduce results. In the future, we aim to extend this study with other alternative normalization procedures and focus on feature repeatability.

### Conclusions

Varying numbers of repeatable breast MRI radiomic features extracted from healthy volunteers were found for each different test–retest strategy. Furthermore, the effects of image preprocessing procedures on the repeatability of radiomic features were found to be different depending on the MRI sequence.

## Supporting information


**Appendix S1**: Supporting Information
