# Supplementary material for: Test–Retest Data for the Assessment of Breast MRI Radiomic Feature Repeatability
Source: J Magn Reson Imaging. 2021 Dec 22;56(2):592–604. doi: 10.1002/jmri.28027 (PMC9544420; doi:10.1002/jmri.28027)

Test-Retest data for the assessment of breast MRI radiomic feature repeatability

Granzier R.W.Y, MD, Ibrahim A, MD, Primakov S, Msc, Keek S.A, Msc, Halilaj I, Msc, Zwanenburg A, PhD, PhD, Engelen S.M.E, MD, PhD, Lobbes M.B.I, MD, PhD, Lambin P, MD, PhD, Woodruff H.C, PhD, Smidt M.L, MD, PhD

Supplementary materials explanation and abbreviations

BFC = Bias Field Correction
DYN = T1-weighted images
VISTA = T2-weigthed images
ADC = ADC maps

The dotted black line in all scatterplots represent the CCC cut off value (CCC>0.90).

Table S1 - acquisition parameters

	RT	TE	ST	FA	WFS	ETL	PS	AM	NoS
T2W	2000	223	2	90	0.28	95	0.79 x 0,79	340, 339	220
T1W	5.30	3.0	2	10	0.39	38	0.36 x 0.36	453, 450	170
DWI	10765	88	3	90	9.46	61	1.01 x 1.01	151, 146	150

Abbreviations: RT, repetition time; TE, echo time; ST, slice thickness; FA, flip angle; WFS, water-fat shift; ETL, echo train-length; PS, pixel spacing; AM, acquisition matrix; NoS, number of slices; T2W, T2-weighted; T1W, T1-weighted; DWI, diffusion-weighted image.

Table S2 - Concordant features across all pairs for the bias field corrected T1-weighted MRI exams, with A: no further pre-processing, B: 32-bin grayscale discretization, C: 64-bin grayscale discretization, D: Z-score normalization, E: Z-score normalization + 32-bin grayscale discretization, and F: Z-score normalization + 64-bin grayscale discretization

	A	B	C	D	E	F
**Number of concordant features**	**8 (8.8%)**	**10 (11.0%)**	**8 (8.8%)**	**4 (4.4%)**	**10 (11.0%)**	**8 (8.8%)**
firstorder_Skewness	x	x	x	x	x	x
firstorder_Uniformity		x	x		x	x
glrlm_GrayLevelNonUniformity	x	x	x		x	x
glrlm_GrayLevelNonUniformityNormalized		x	x		x	x
glrlm_RunLengthNonUniformity	x		x			x
glszm_GrayLevelNonUniformity	x		x	x		
glszm_LargeAreaHighGrayLevelEmphasis		x			x	
glszm_SizeZoneNonUniformity	x			x		
gldm_DependenceEntropy		x			x	
gldm_DependenceNonUniformity	x	x			x	
gldm_GrayLevelNonUniformity	x	x		x	x	x
ngtdm_Busyness		x	x		x	x
ngtdm_Coarseness	x	x	x		x	x

Table S3 - Concordant features across all pairs for the bias field corrected T2-weighted MRI exams, with A: no further pre-processing, B: 32-bin grayscale discretization, C: 64-bin grayscale discretization, D: Z-score normalization, E: Z-score normalization + 32-bin grayscale discretization, and F: Z-score normalization + 64-bin grayscale discretization.

	**A**	**B**	**C**	**D**	**E**	**D**
**Number of stable features**	**0 (0.0%)**	**2 (2.2%)**	**1 (1.1%)**	**26 (28.6%)**	**6 (6.6%)**	**5 (5.5%)**
firstorder_10Percentile				x	x	x
firstorder_InterquartileRange				x	x	x
firstorder_Kurtosis			x			
firstorder_MeanAbsoluteDeviation				x	x	x
firstorder_RobustMeanAbsoluteDeviation				x	x	x
glcm_Contrast				x		
glcm_DifferenceAverage				x		
glcm_DifferenceEntropy				x		
glcm_DifferenceVariance				x		
glcm_JointEntropy				x		
glcm_Idm				x		
glcm_Idmn				x		
glcm_Id				x		
glcm_Idn				x		
glcm_InverseVariance				x		
glcm_SumEntropy				x		
glrlm_GrayLevelNonUniformity		x	x		x	x
glrlm_RunPercentage				x		
glrlm_RunVariance				x		
gldm_DependenceEntropy				x		
gldm_DependenceNonUniformity				x		
gldm_DependenceNonUniformityNormalized				x		
gldm_DependenceVariance				x		
gldm_GrayLevelNonUniformity				x		
gldm_LargeDependenceEmphasis				x		
gldm_SmallDependenceEmphasis				x		
gldm_SmallDependenceHighGrayLevelEmphasis				x		
gldm_SmallDependenceLowGrayLevelEmphasis		x			x	
ngtdm_Complexity				x		

Figure S1 - T1-weighted images with and without BFC without further image pre-processing: scatterplots of pairwise CCC values for all features wherein orange represents the use of all pairwise comparisons, blue represents the pairwise comparisons between MRI exams scanned on Date 1 and green represent the pairwise comparisons between MRI exams scanned on Date 2

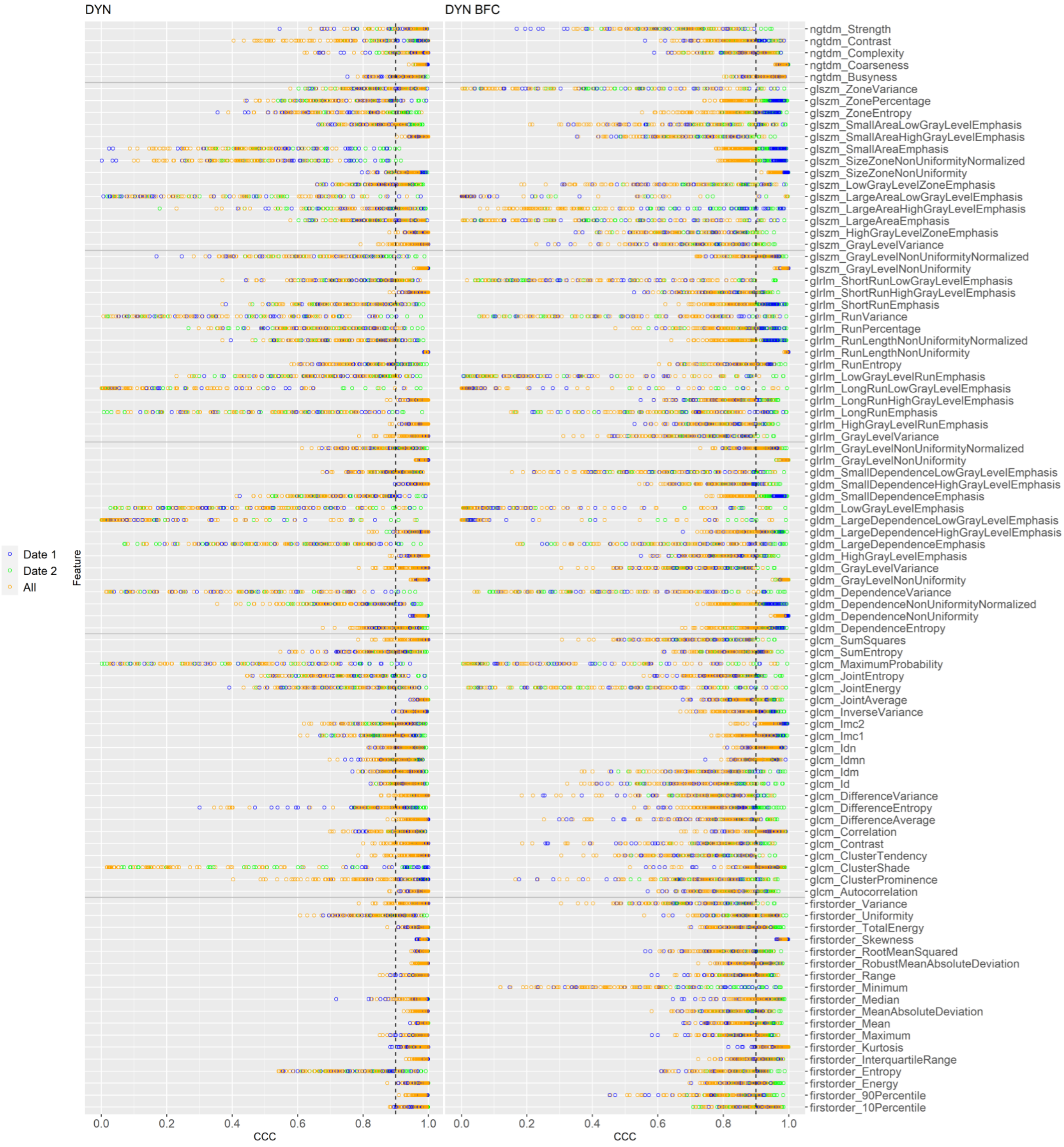

Figure S2 - T1-weighted images with and without BFC with 32-bin grayscale discretization: scatterplots of pairwise CCC values for all features wherein orange represents the use of all pairwise comparisons, blue represents the pairwise comparisons between MRI exams scanned on Date 1 and green represent the pairwise comparisons between MRI exams scanned on Date 2

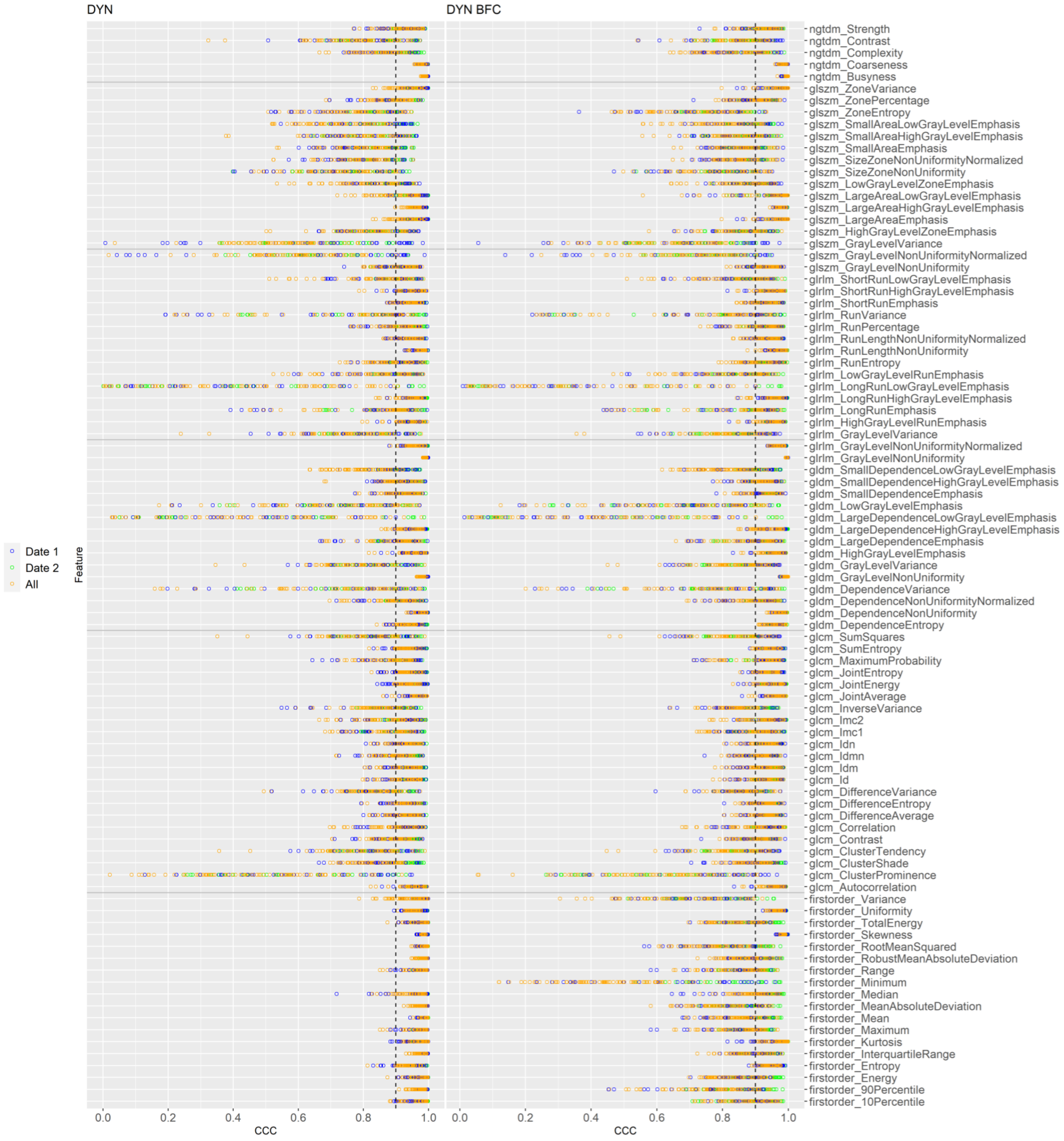

Figure S3 - T1-weighted images with and without BFC with 32-bin grayscale discretization: scatterplots of pairwise CCC values for all features wherein orange represents the use of all pairwise comparisons, blue represents the pairwise comparisons between MRI exams scanned on Date 1 and green represent the pairwise comparisons between MRI exams scanned on Date 2

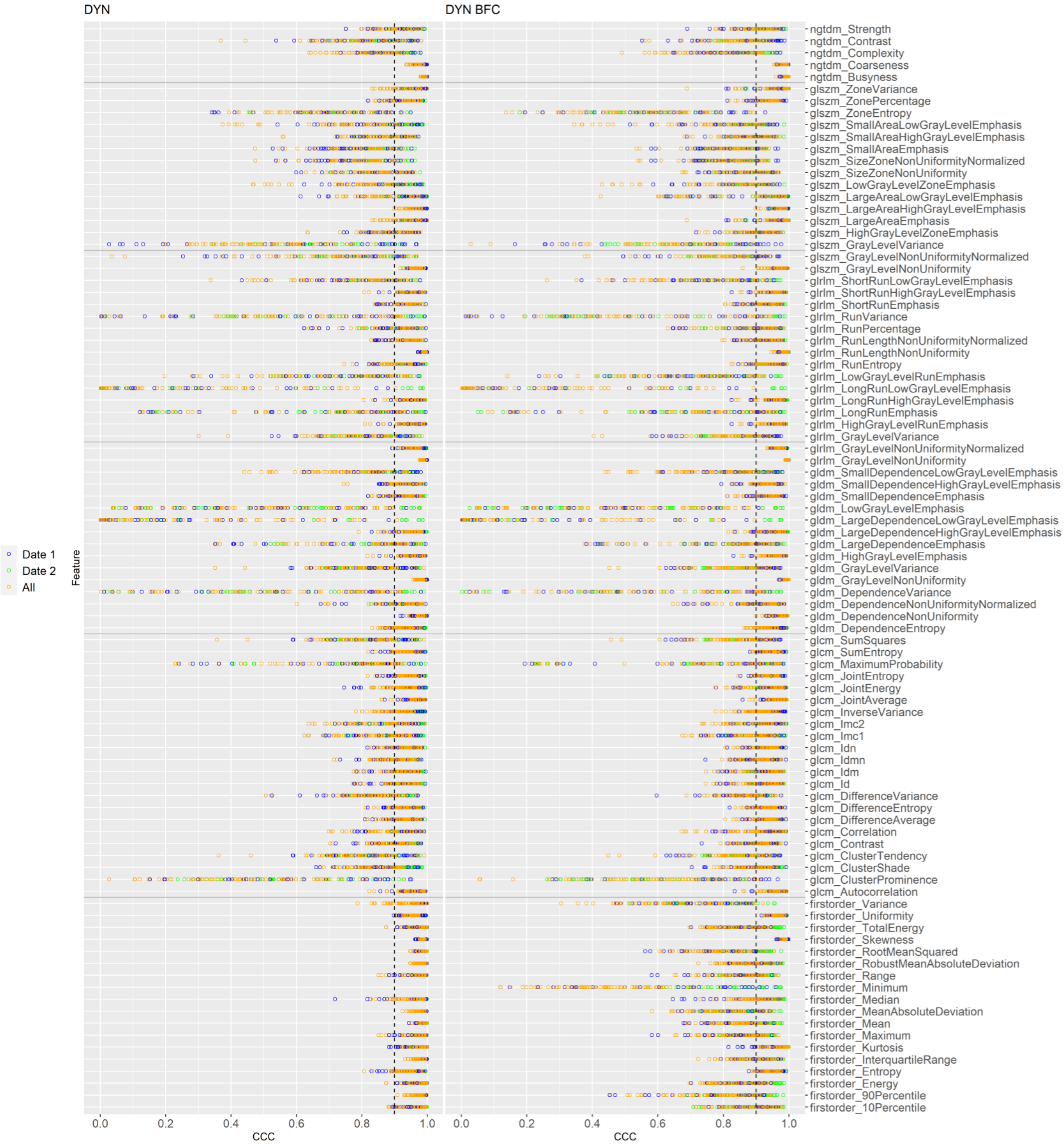

Figure S4 - T1-weighted images with and without BFC with z-score normalization: scatterplots of pairwise CCC values for all features wherein orange represents the use of all pairwise comparisons, blue represents the pairwise comparisons between MRI exams scanned on Date 1 and green represent the pairwise comparisons between MRI exams scanned on Date 2

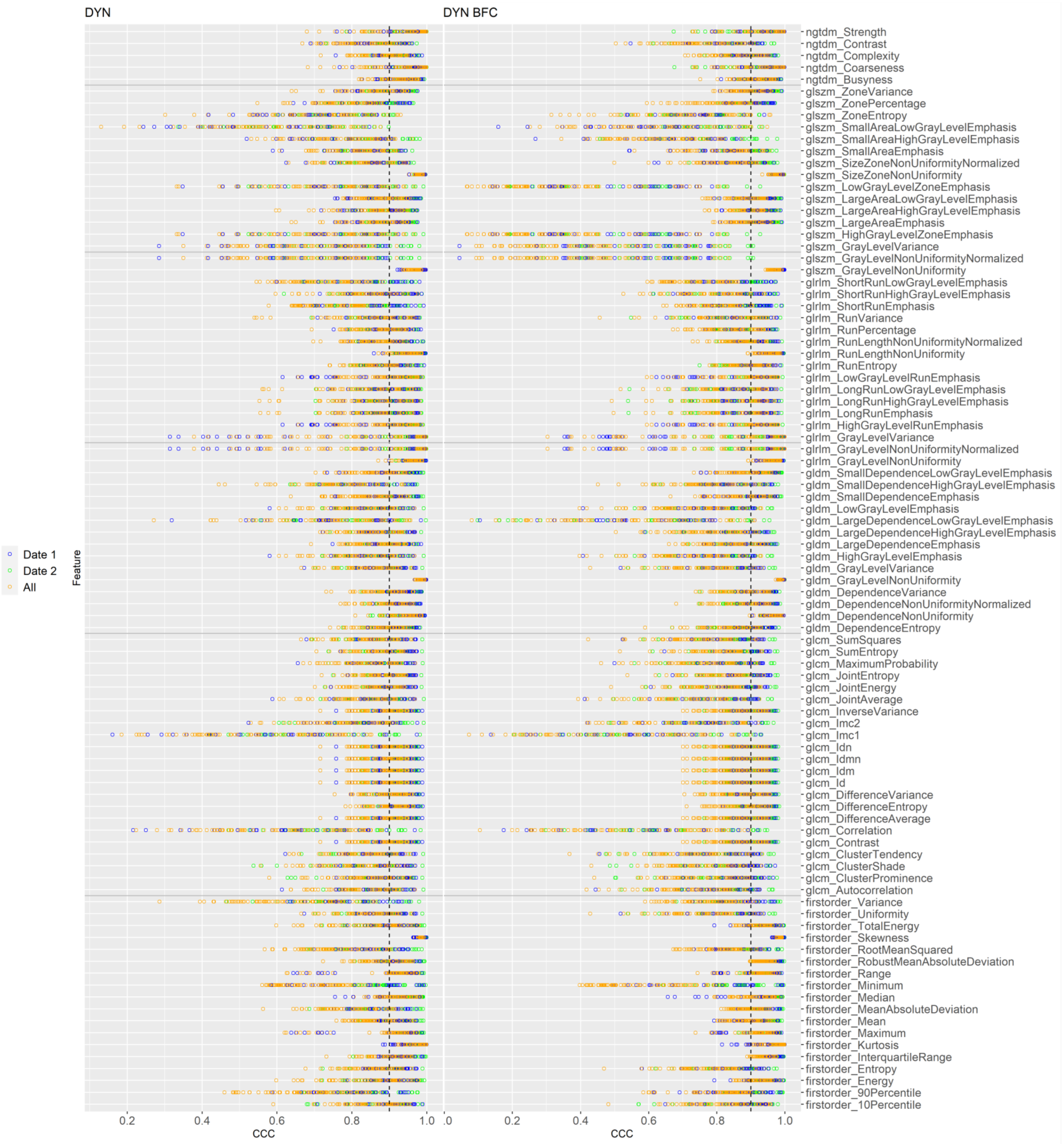

Figure S5 - T1-weighted images with and without BFC with z-score normalization and 32-bin grayscale discretization: scatterplots of pairwise CCC values for all features wherein orange represents the use of all pairwise comparisons, blue represents the pairwise comparisons between MRI exams scanned on Date 1 and green represent the pairwise comparisons between MRI exams scanned on Date 2

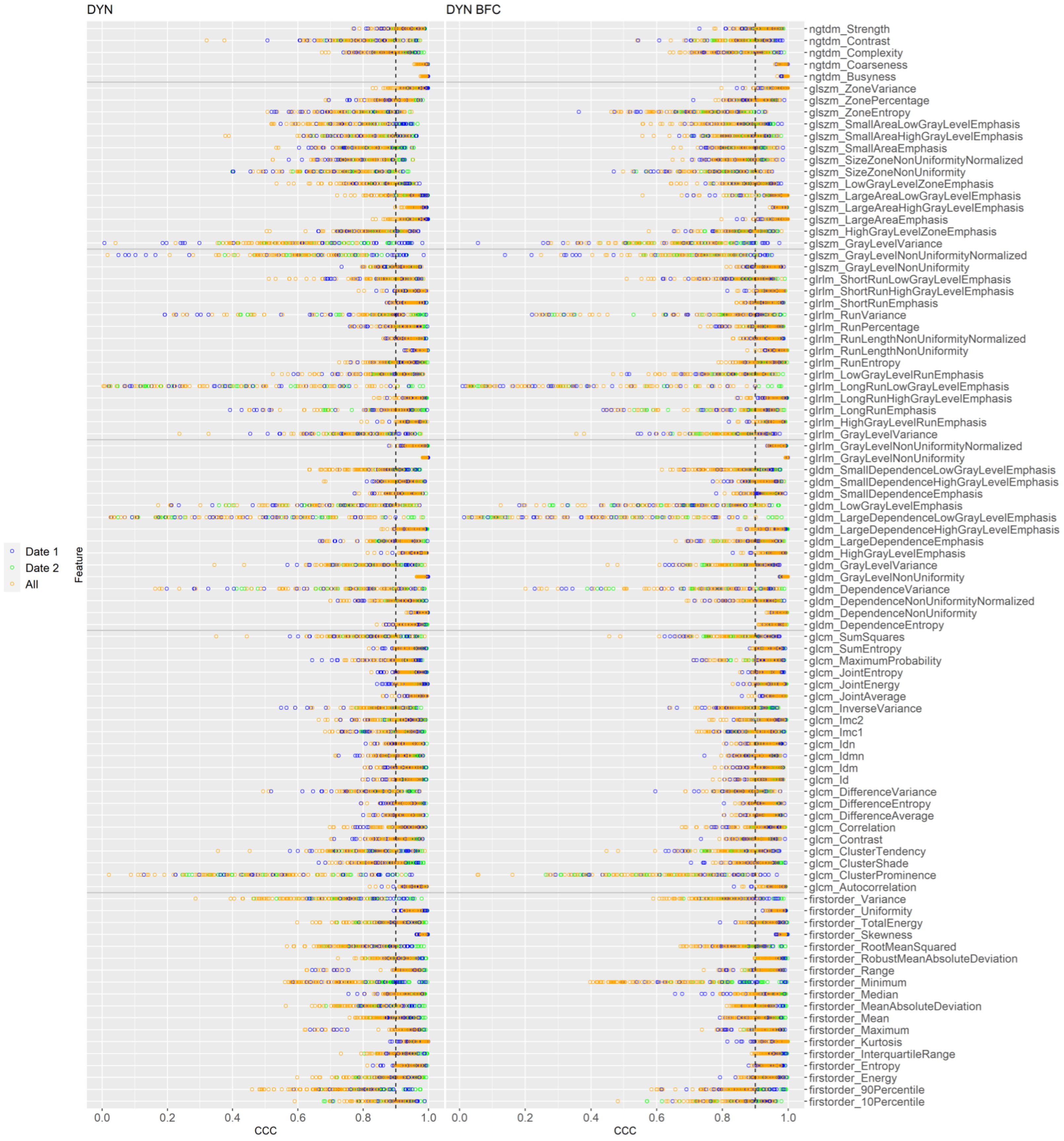

Figure S6 - T1-weighted images with and without BFC with z-score normalization and 64-bin grayscale discretization: scatterplots of pairwise CCC values for all features wherein orange represents the use of all pairwise comparisons, blue represents the pairwise comparisons between MRI exams scanned on Date 1 and green represent the pairwise comparisons between MRI exams scanned on Date 2

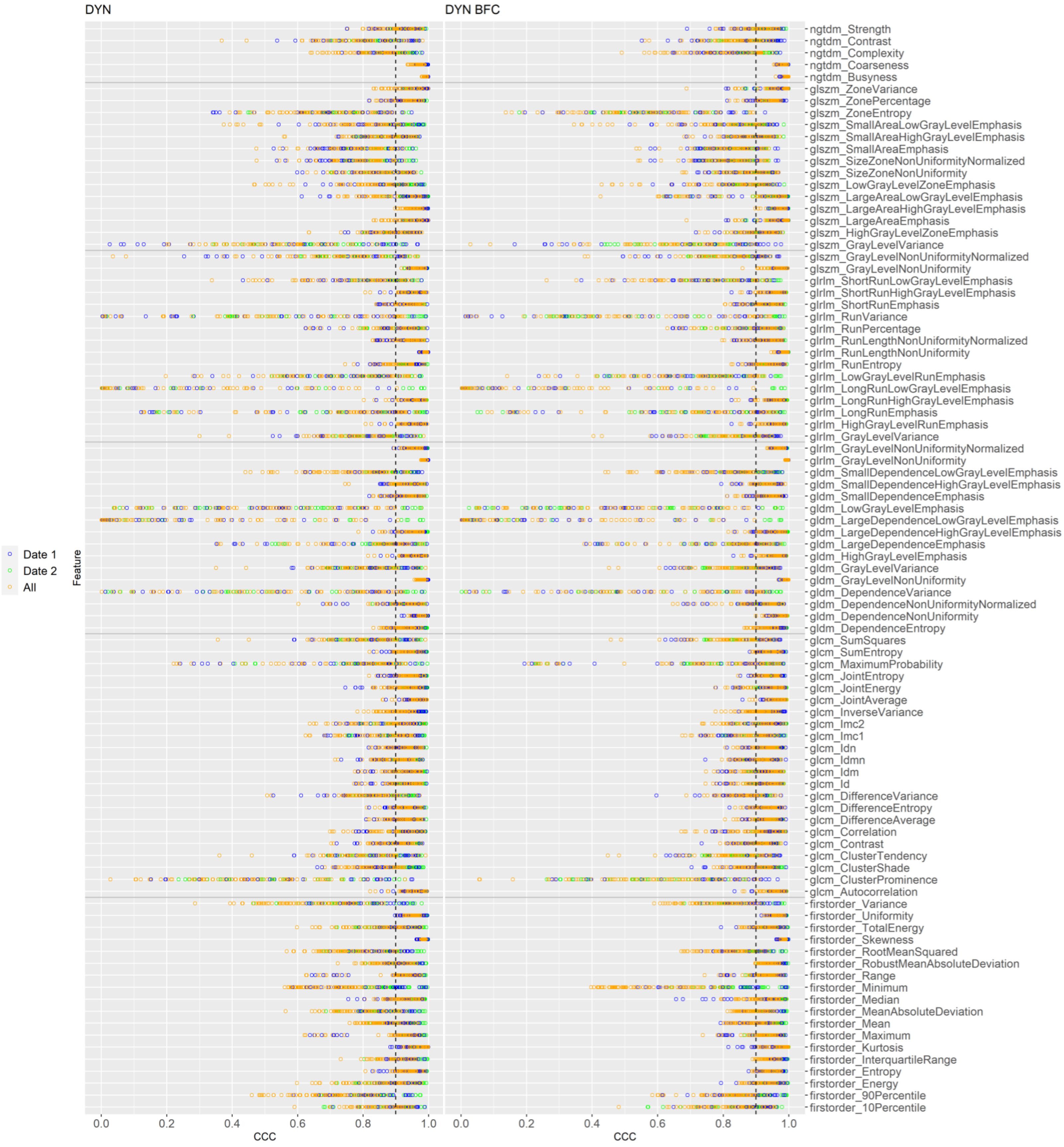

Figure S7 - T2-weighted images with and without BFC without further pre-processing: scatterplots of pairwise CCC values for all features wherein orange represents the use of all pairwise comparisons, blue represents the pairwise comparisons between MRI exams scanned on Date 1 and green represent the pairwise comparisons between MRI exams scanned on Date 2

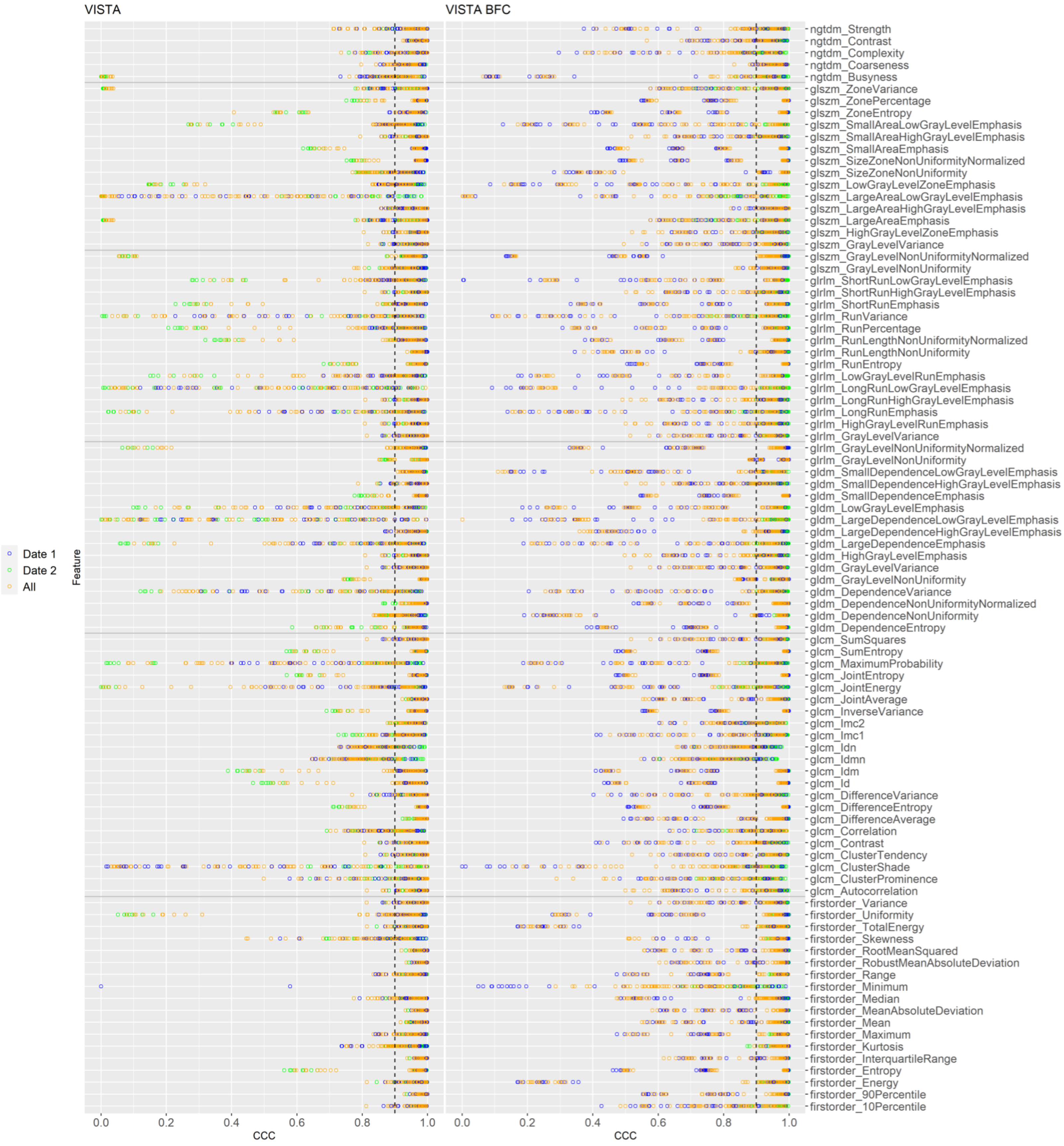

Figure S8 - T2-weighted images with and without BFC with 32-bin grayscale discretization: scatterplots of pairwise CCC values for all features wherein orange represents the use of all pairwise comparisons, blue represents the pairwise comparisons between MRI exams scanned on Date 1 and green represent the pairwise comparisons between MRI exams scanned on Date 2

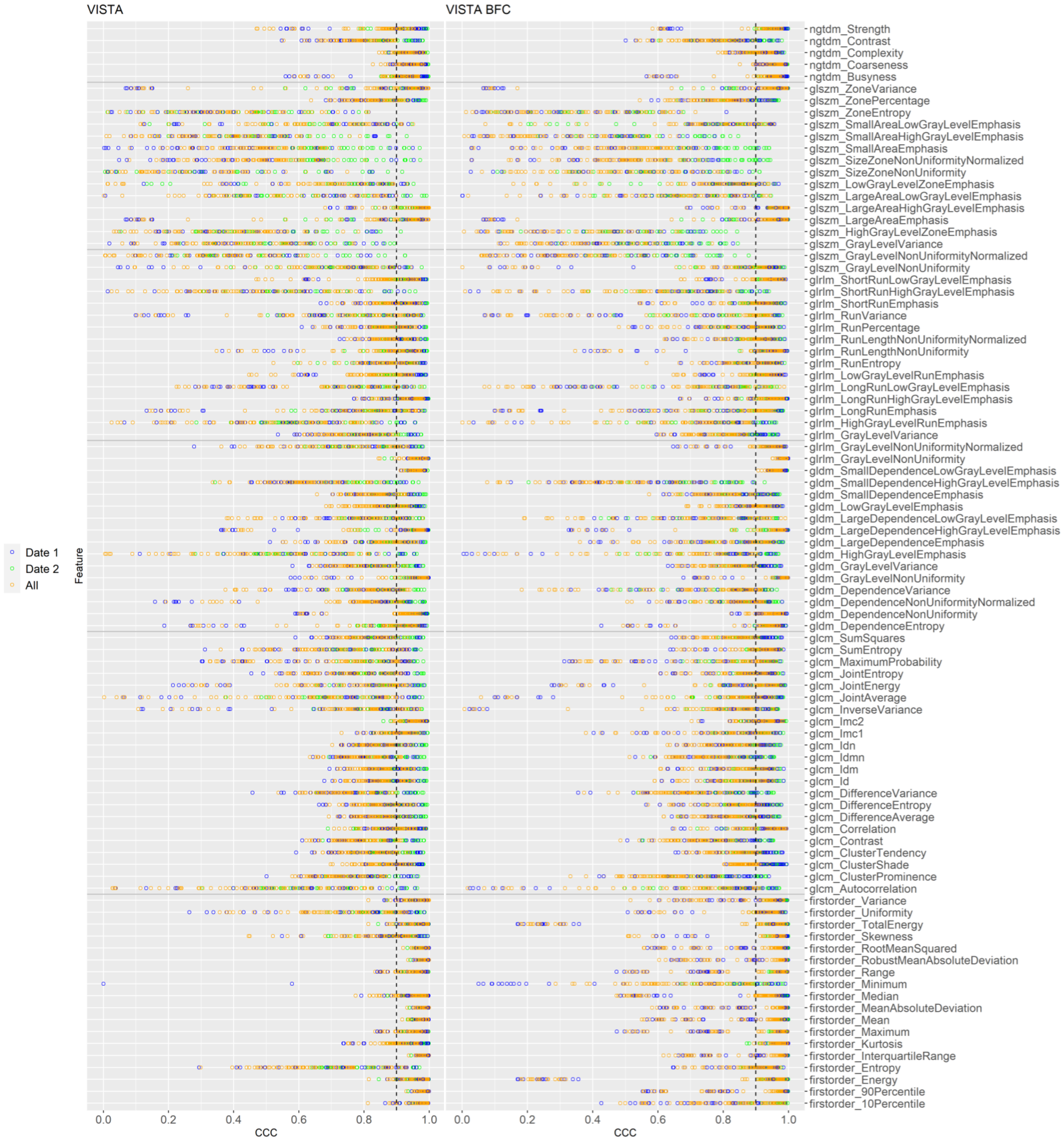

Figure S9 - T2-weighted images with and without BFC with 64-bin grayscale discretization: scatterplots of pairwise CCC values for all features wherein orange represents the use of all pairwise comparisons, blue represents the pairwise comparisons between MRI exams scanned on Date 1 and green represent the pairwise comparisons between MRI exams scanned on Date 2

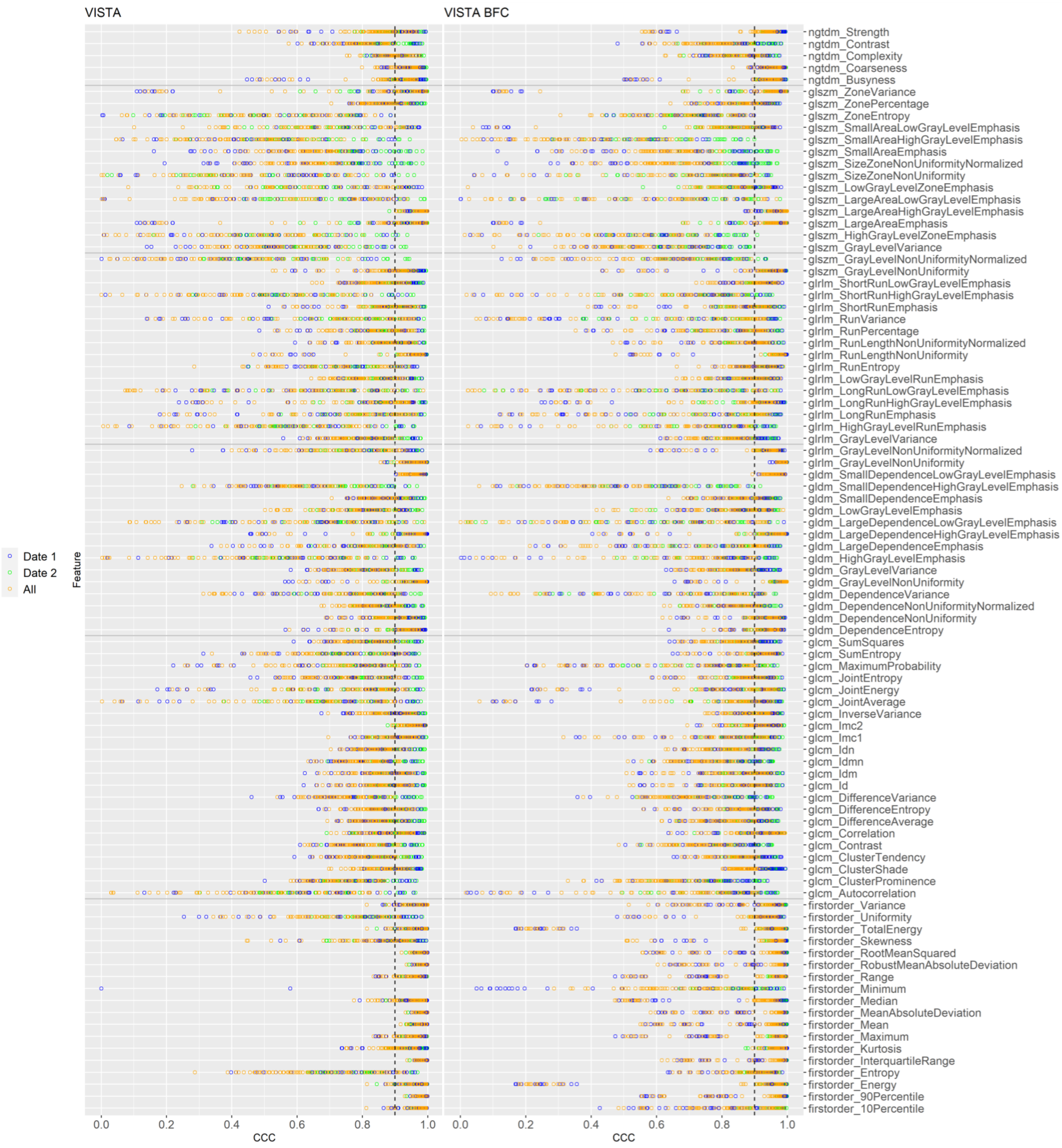

Figure S10 - T2-weighted images with and without BFC with z-score normalization: scatterplots of pairwise CCC values for all features wherein orange represents the use of all pairwise comparisons, blue represents the pairwise comparisons between MRI exams scanned on Date 1 and green represent the pairwise comparisons between MRI exams scanned on Date 2

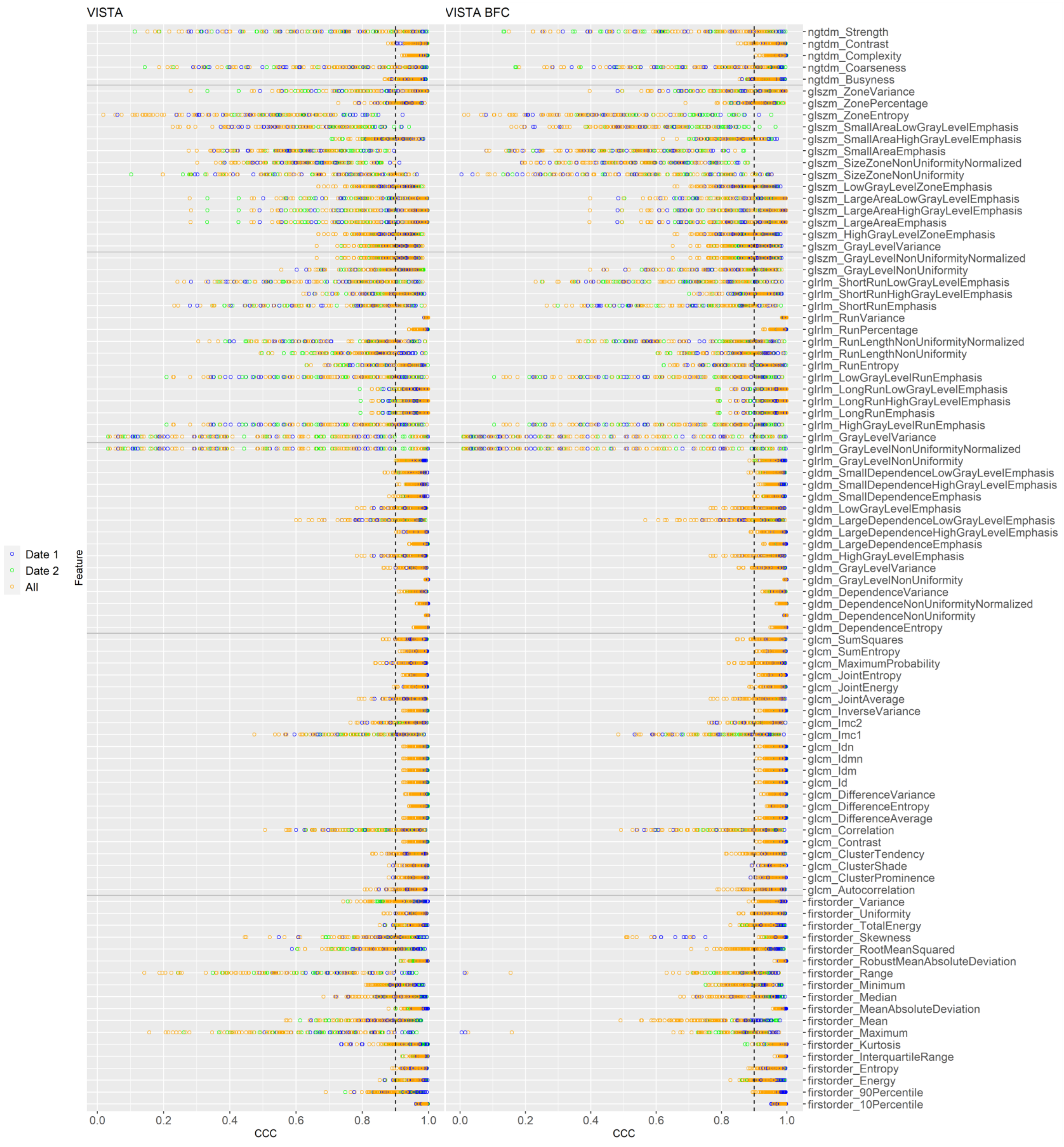

Figure S11 - T2-weighted images with and without BFC with z-score normalization and 32-bin grayscale discretization: scatterplots of pairwise CCC values for all features wherein orange represents the use of all pairwise comparisons, blue represents the pairwise comparisons between MRI exams scanned on Date 1 and green represent the pairwise comparisons between MRI exams scanned on Date 2

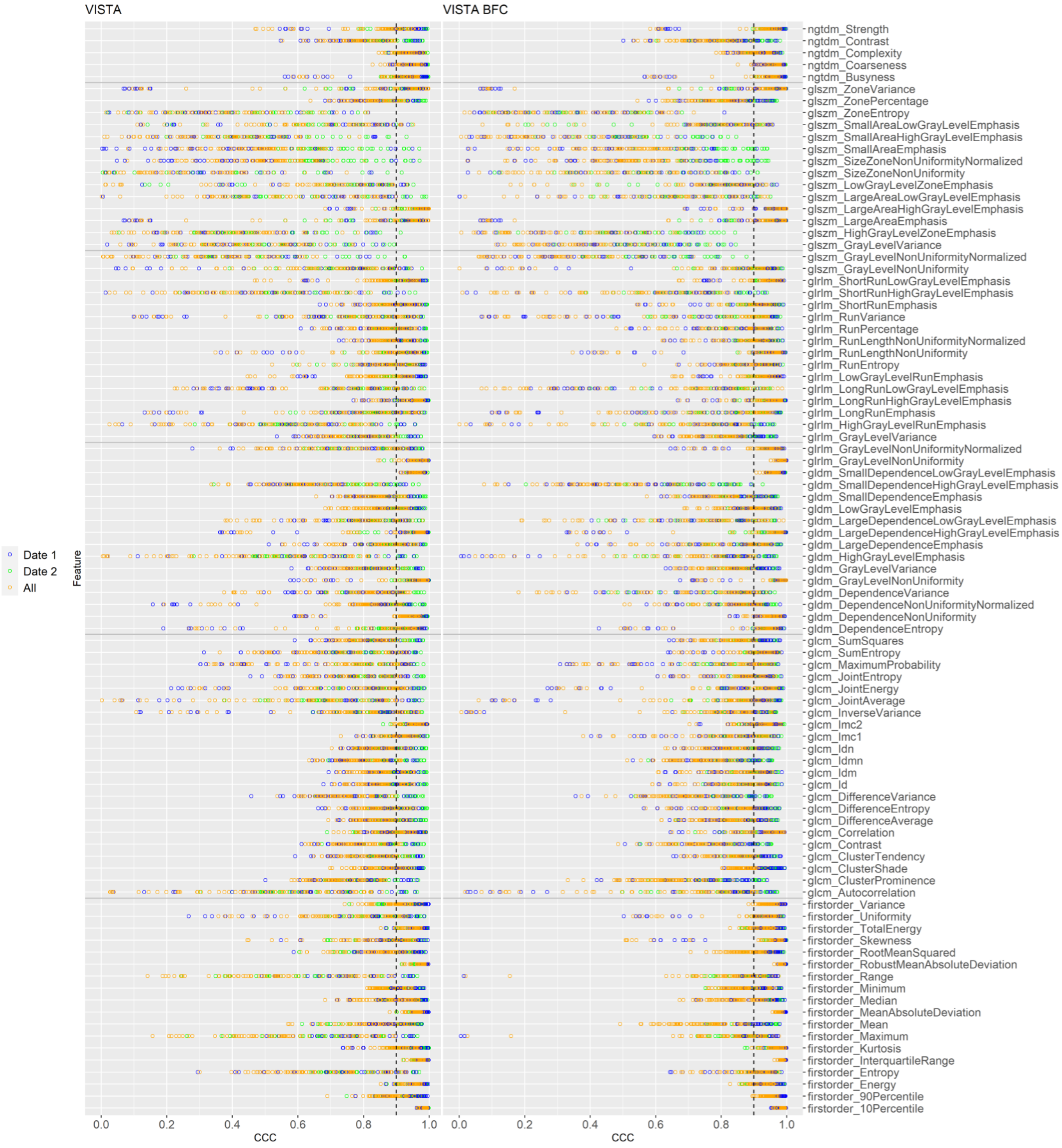

Figure S12 - T2-weighted images with and without BFC with z-score normalization and 64-bin grayscale discretization: scatterplots of pair-wise CCC values for all features wherein orange represents the use of all pairwise comparisons, blue represents the pairwise comparisons between MRI exams scanned on Date 1 and green represent the pairwise comparisons between MRI exams scanned on Date 2

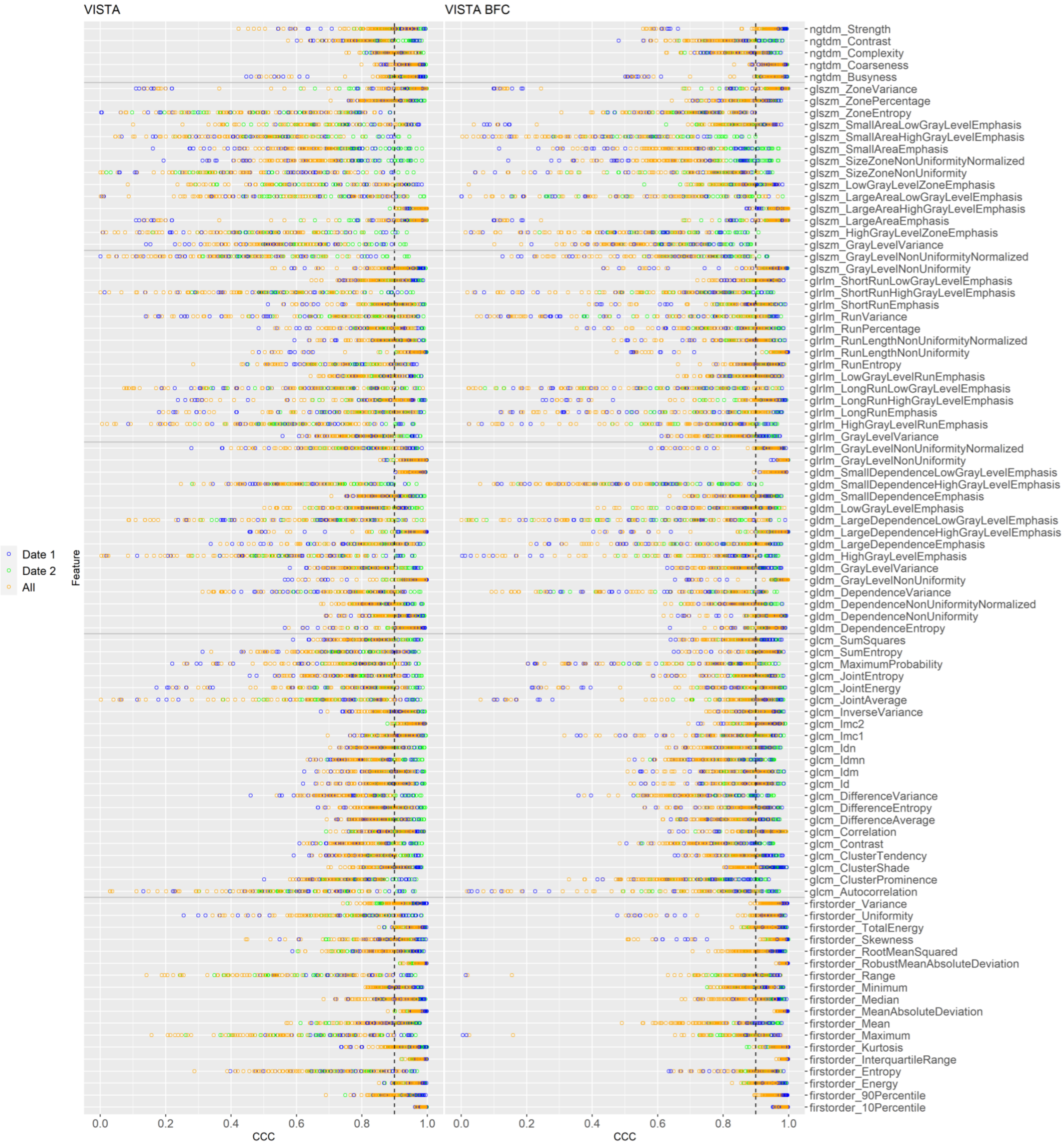

Figure S13 - ADC maps with and without BFC without further pre-processing: scatterplots of pairwise CCC values for all features wherein orange represents the use of all pairwise comparisons, blue represents the pairwise comparisons between MRI exams scanned on Date 1 and green represent the pairwise comparisons between MRI exams scanned on Date 2

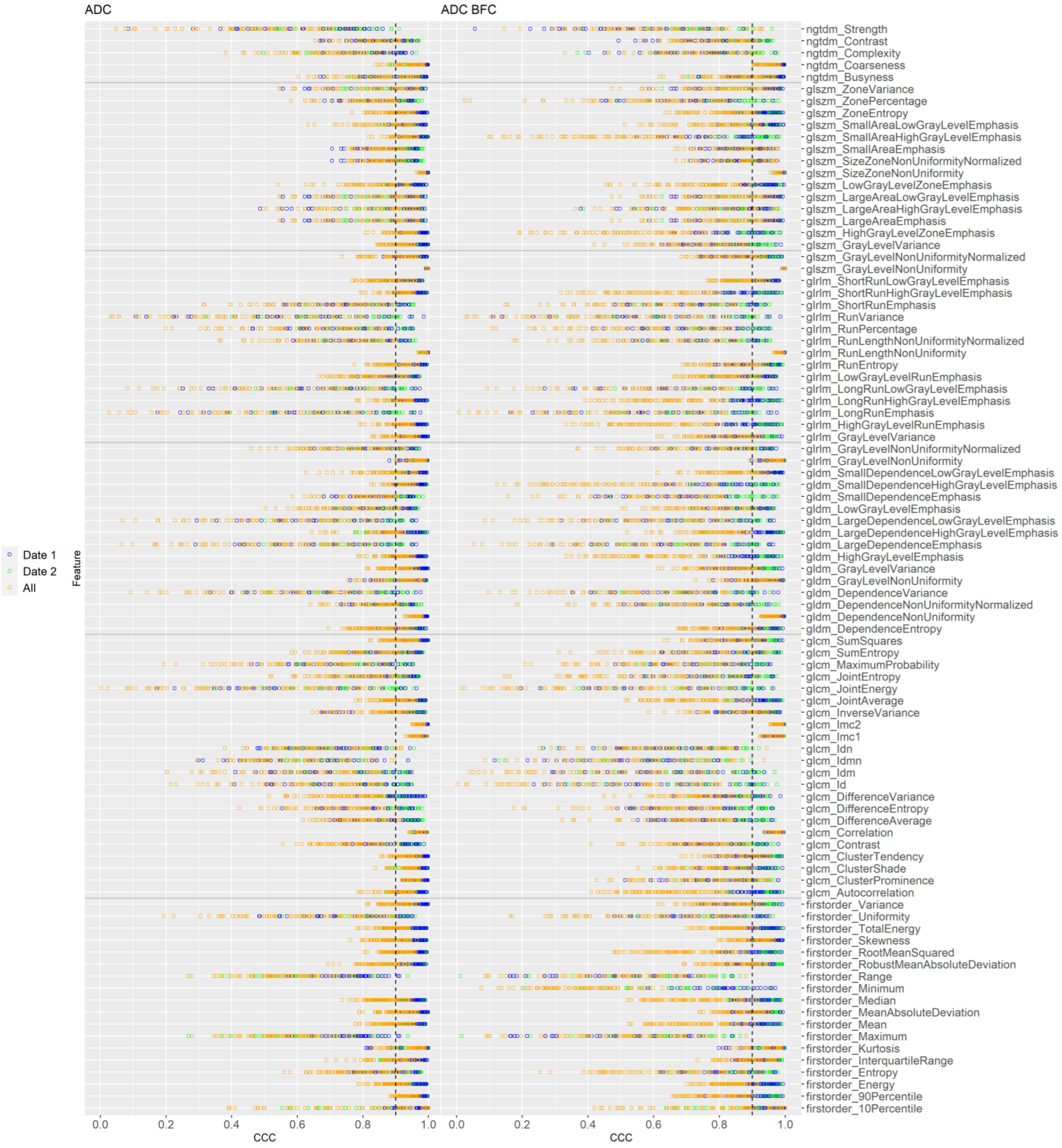

Figure S14 - ADC maps with and without BFC with 32-bin grayscale discretization: scatterplots of pairwise CCC values for all features wherein orange represents the use of all pairwise comparisons, blue represents the pairwise comparisons between MRI exams scanned on Date 1 and green represent the pairwise comparisons between MRI exams scanned on Date 2

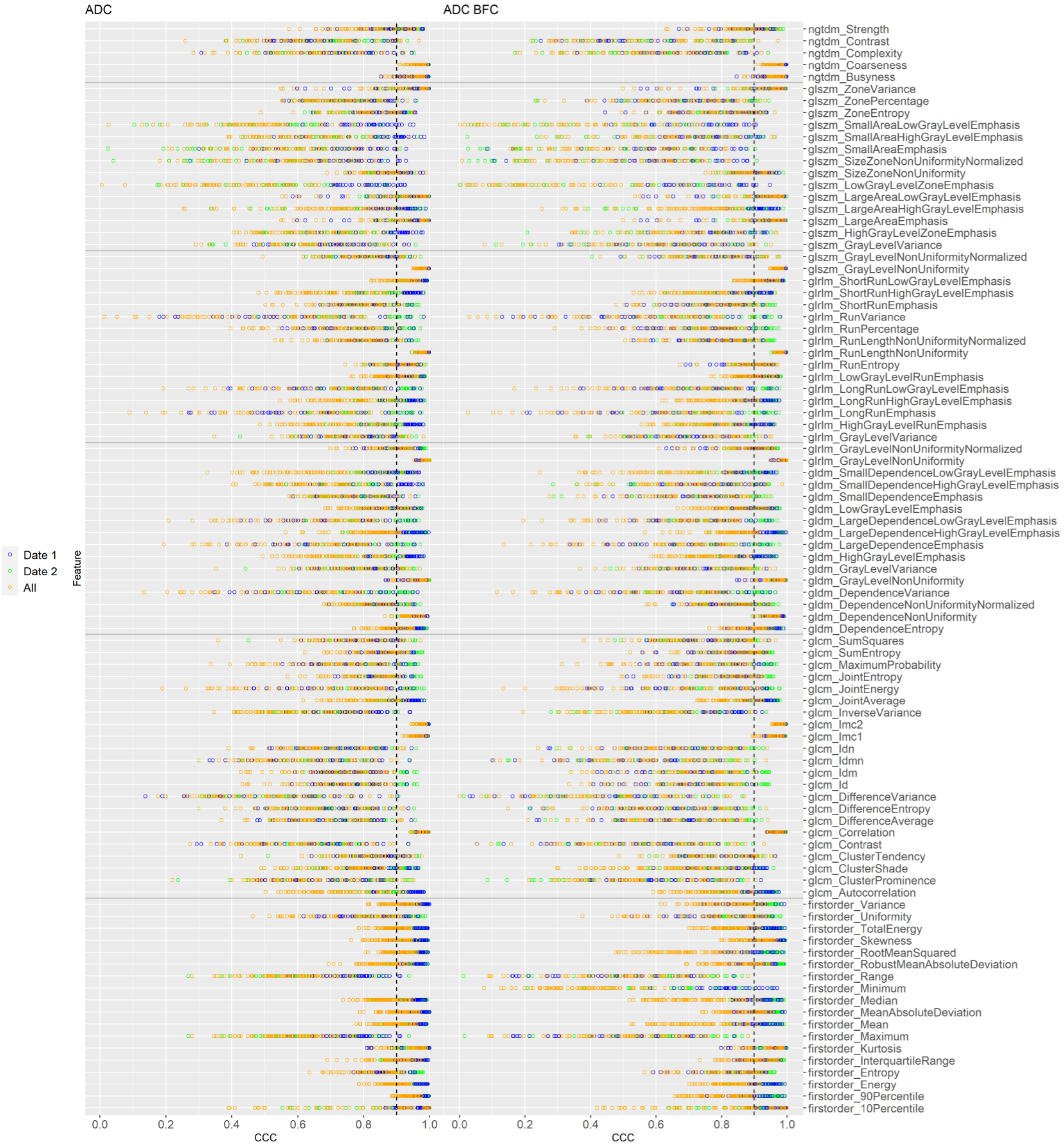

Figure S15 - ADC maps with and without BFC with 64-bin grayscale discretization: scatterplots of pairwise CCC values for all features wherein orange represents the use of all pairwise comparisons, blue represents the pairwise comparisons between MRI exams scanned on Date 1 and green represent the pairwise comparisons between MRI exams scanned on Date 2

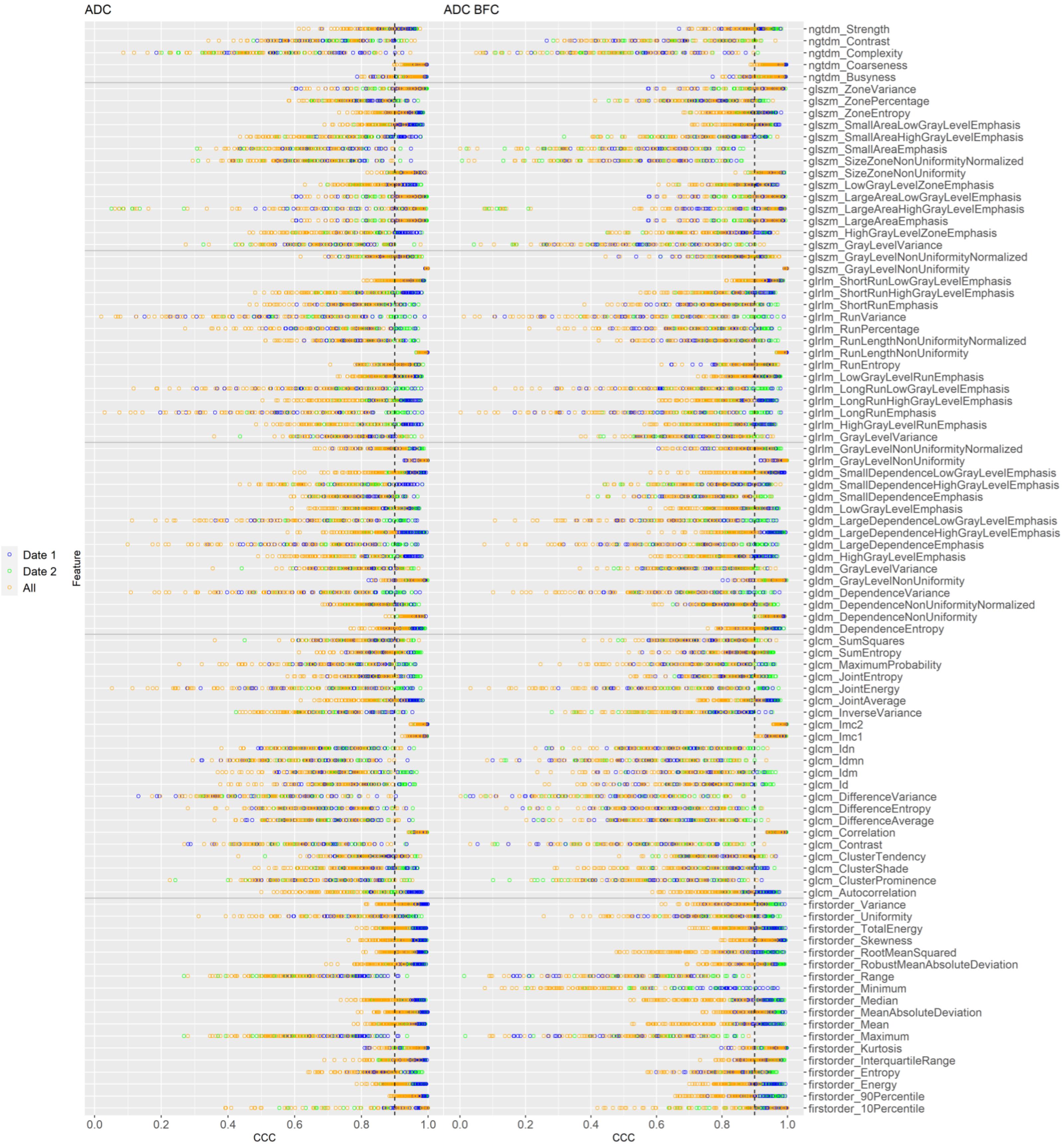

Supplement: Supplementary file 1 — Appendix S1: Supporting Information [file JMRI-56-592-s001.pdf]
